# *Streptomyces venezuelae* NRRL B-65442: genome sequence of a model strain used to study morphological differentiation in filamentous actinobacteria

**DOI:** 10.1093/jimb/kuab035

**Published:** 2021-06-08

**Authors:** Juan Pablo Gomez-Escribano, Neil A Holmes, Susan Schlimpert, Maureen J Bibb, Govind Chandra, Barrie Wilkinson, Mark J Buttner, Mervyn J Bibb

**Affiliations:** Department of Molecular Microbiology, John Innes Centre, Norwich Research Park, Norwich NR4 7UH, UK; Department of Molecular Microbiology, John Innes Centre, Norwich Research Park, Norwich NR4 7UH, UK; Department of Molecular Microbiology, John Innes Centre, Norwich Research Park, Norwich NR4 7UH, UK; Department of Molecular Microbiology, John Innes Centre, Norwich Research Park, Norwich NR4 7UH, UK; Department of Molecular Microbiology, John Innes Centre, Norwich Research Park, Norwich NR4 7UH, UK; Department of Molecular Microbiology, John Innes Centre, Norwich Research Park, Norwich NR4 7UH, UK; Department of Molecular Microbiology, John Innes Centre, Norwich Research Park, Norwich NR4 7UH, UK; Department of Molecular Microbiology, John Innes Centre, Norwich Research Park, Norwich NR4 7UH, UK

**Keywords:** Streptomyces venezuelae, Genome sequence, Spore pigment, CRISPR Cas9, pSVJI1, Plasmid curing

## Abstract

For over a decade, *Streptomyces venezuelae* has been used to study the molecular mechanisms that control morphological development in streptomycetes and is now a well-established model strain. Its rapid growth and ability to sporulate in a near-synchronised manner in liquid culture, unusual among streptomycetes, greatly facilitates the application of modern molecular techniques such as ChIP-seq and RNA-seq, as well as time-lapse fluorescence imaging of the complete *Streptomyces* life cycle. Here we describe a high-quality genome sequence of our isolate of the strain (Northern Regional Research Laboratory [NRRL] B-65442) consisting of an 8.2 Mb chromosome and a 158 kb plasmid, pSVJI1, which had not been reported previously. Surprisingly, while NRRL B-65442 yields green spores on MYM agar, the American Type Culture Collection (ATCC) type strain 10712 (from which NRRL B-65442 was derived) produces grey spores. While comparison of the genome sequences of the two isolates revealed almost total identity, it did reveal a single nucleotide substitution in a gene, *vnz_33525*, involved in spore pigment biosynthesis. Replacement of the *vnz_33525* allele of ATCC 10712 with that of NRRL B-65442 resulted in green spores, explaining the discrepancy in spore pigmentation. We also applied CRISPR-Cas9 to delete the essential *parB* of pSVJI1 to cure the plasmid from the strain without obvious phenotypic consequences.

## Introduction

*Streptomyces* species are high G + C (∼70 mol% GC) Gram-positive bacteria characterised by filamentous growth and a complex life cycle: Drought-resistant spores germinate under appropriate environmental conditions to produce a vegetative mycelium that develops aerial hyphae and eventually spores. The entire developmental programme is tightly controlled by a complex regulatory network (Bush et al., 2015). Streptomycetes are also very prolific producers of specialised metabolites with a wide range of biological activities, including antimicrobials, antihelminthics, antitumour compounds, immunosuppressants, and siderophores.

*Streptomyces venezuelae* was first described as a producer of the antimicrobial compound chloromycetin (Ehrlich et al., 1947) (later renamed chloramphenicol; Smadel, 1949) after isolation from a soil sample from Caracas, Venezuela (Ehrlich et al., 1948). This first isolate was designated the type strain of the species and deposited at the American Type Culture Collection (ATCC) as strain 10712 (Ehrlich et al., 1949). Other *Streptomyces* species were later isolated as producers of chloramphenicol and some identified as *S. venezuelae* (see Supplementary Table S2 for details).

One of the most striking characteristics of *S. venezuelae* is its unusual ability to sporulate in liquid culture (Glazebrook et al., 1990). This has greatly facilitated the application of global omics techniques such as DNA microarrays (Pullan et al., 2011; Bibb et al., 2012), ChIP-seq and RNA-seq (Al-Bassam et al., 2014; Tschowri et al., 2014; Bush et al., 2013, 2016, 2019; Haist et al., 2020), and time-lapse fluorescence microscopy (Donczew et al., 2016; Schlimpert et al., 2016, 2017; Fröjd & Flärdh, 2019) to study *Streptomyces* development. The adoption of *S. venezuelae* also led directly to the discovery of exploratory growth, a previously undescribed mode of *Streptomyces* development in which non-branching vegetative hyphae rapidly transverse solid surfaces (Jones et al., 2017). Consequently, *S. venezuelae* is now considered to be a model strain for the study of morphological differentiation in *Streptomyces* (Bush et al., 2015; Chater, 2016).

The *S. venezuelae* strain used at the John Innes Centre (JIC) was obtained from Diversa Corporation (San Diego, USA) in 2003 and described as strain ATCC 10712; the company had in turn obtained it from the Vining laboratory (Doull et al., 1985). In addition to chloramphenicol (Fernández-Martínez et al., 2014), this strain also produces the polyketide antibiotic jadomycin B (Doull et al., 1993, 1994; Jakeman et al., 2006), which also has antitumour activity (Hall et al., 2015), and its congeners (Robertson et al., 2015), as well as the biaryl polyketide venemycin (Thanapipatsiri et al., 2016), the non-ribosomal peptide watasemycin (Inahashi et al., 2017), the γ-aminobutyrate-derived gaburedins (Sidda et al., 2013), and the indole arcyriaflavin (Mervyn Bibb, unpublished data); it also has the potential to produce a novel lanthipeptide, venezuelin (Goto et al., 2010). At the same time, Diversa Corporation also kindly provided a genome sequence of the strain that had been obtained by whole-genome shotgun Sanger sequencing (GenBank Accession No. FR845719). Although of high quality, the sequence contained a number of gaps (Pullan et al., 2011). Recent advances in DNA sequencing, particularly the advent of third-generation technologies such as Pacific Biosciences SMRT technology (PacBio), now allow the affordable generation of genome assemblies with single-contigs per replicon and with 99.999% accuracy (Gomez-Escribano et al., 2015). We thus set out to establish an improved high-quality genome sequence for the JIC isolate (now designated Northern Regional Research Laboratory [NRRL] B-65442) that closed the gaps in the Diversa sequence of the same strain and also allowed us to assess plasmid content.

## Methods

*S. venezuelae* strains were cultivated in liquid or agar MYM (Stuttard, 1982). Apramycin and nalidixic acid were purchased from Sigma. *Escherichia coli* DH5α was used as a general-purpose cloning host following established procedures (Sambrook et al., 1989). *E. coli* ET12567/pUZ8002 was used as the donor strain in the *E. coli*-*Streptomyces* conjugations following established methods (Kieser et al., 2000). For details of genome sequencing and allele replacement, see the Supplementary material.

## Results

### A Difference in Spore Pigmentation Results in Designation of the JIC Isolate of *S. venezuelae* as NRRL B-65442

**Fig. 1:**
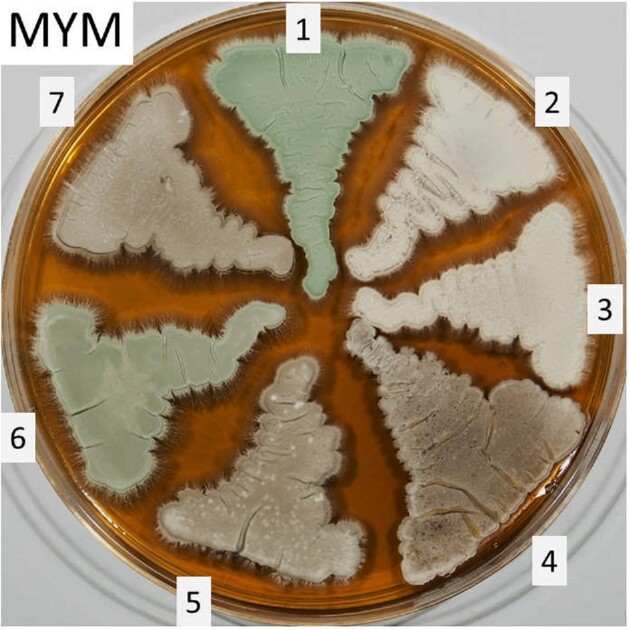
Sporulation phenotypes of different *S. venezuelae* strains grown on MYM agar for 7 days at 30°C. 1, NRRL B-65442; 2, NRRL B-902; 3, ATCC 10595; 4, NRRL 2277; 5, ATCC 10712; 6, Sven_Dalhousie; 7, strain 13 s. With the exception of NRRL B-65442 (the JIC strain), the NRRL and ATCC isolates were obtained from the respective culture collections; Sven_Dalhousie (formerly designated ATCC 10712) and strain 13 s (another *S. venezuelae* isolate used by the Vining laboratory (Ahmed & Vining, 1983)) were kindly provided by David Jakeman from the Vining collection. See the Supplementary material for more information about the origin of the different strains. The original RAW file is available from the authors upon request.

In the course of other work, we obtained *S. venezuelae* ATCC 10712 directly from ATCC and were surprised to find that while our isolate (obtained from the Vining laboratory as ATCC 10712), and a recently obtained culture from the Vining collection (kindly provided by David Jakeman, Dalhousie University, Canada), produced green spores when cultivated on MYM agar, the ATCC strain, and indeed isolates of *S. venezuelae* from other culture collections, produced grey spores (Fig. 1; see Supplementary Fig. S1). All of these strains were shown to produce chloramphenicol (see Supplementary Fig. S2). Given this phenotypic difference, and to prevent future confusion, we deposited the JIC isolate at the Agricultural Research Service Culture Collection (the NRRL collection) as *Streptomyces venezuelae* NRRL B-65442 and at the Leibniz Institute DSMZ-German Collection of Microorganisms and Cell Cultures (DSMZ) as DSM 112328.

### Genome Sequencing of *S. venezuelae* NRRL B-65442

We obtained a very comprehensive and high-quality genome assembly using Pacific Biosciences SMRT technology; sequence data (average coverage of 150×) was assembled with HGAP.3 in two contigs: unitig_0 of 8,208,916 bp with an average coverage of 145×, and unitig_1 of 144,576 bp with an average coverage of 743× (Supplementary Table S3). We had shown previously that such assemblies do not contain all of the data produced during sequencing (Gomez-Escribano et al., 2015). Consequently, blastN searches with the ends of the two contigs were used to identify additional sequences in the data set that were added to the assemblies using GAP4 (Staden et al., 1999). The final PacBio assembled contigs were 8,211,324 bp and 158,122 bp in length corresponding to the expected 8.2 Mb chromosome and a 158 kb previously unidentified putative extra-chromosomal replicon.

The chromosomal contig still lacked 7511 bp at the left end and 3663 bp at the right end when compared with the Diversa sequence (GenBank Accession No. FR845719). To extend the PacBio contigs as much as possible, we undertook a chromosome-walking approach; we designed oligonucleotides based on the Diversa sequence to use as PCR-primers to cover all known remaining sequence at each end of the chromosome. The PCR products were sequenced by Sanger sequencing with the same primers used for the PCR amplification, and the Sanger reads were added to the PacBio contig with GAP4. The final extended chromosome sequence consisted of 8,222,198 bp (because of difficulty in designing primers at the very ends of the previous sequence, our extended sequence is 78 bp shorter at the left end and 229 bp at the right end than the Diversa assembly).

The final chromosome and likely plasmid sequences were deposited at NCBI with accession numbers CP018074.1 and CP018075.1, respectively (assembly accession GCA_001886595.1), and are also available at StrepDB (http://strepdb.streptomyces.org.uk/).

The chromosome has a base composition of 72.5 mol%GC and contains 7141 protein coding sequences, seven rRNA gene clusters, and 70 tRNA genes. The putative plasmid has a GC content of 70.1 mol%GC and contains 163 protein coding sequences. Analysis of the chromosome sequence using antiSMASH (Blin et al., 2019) followed by manual curation revealed 34 likely natural product gene clusters that included those for the seven compounds known to be made, or with the potential to be made, by NRRL B-65442: chloramphenicol (Fernández-Martínez et al., 2014), jadomycin B (Jakeman et al., 2006), venemycin (Thanapipatsiri et al., 2016), watasemycin (Inahashi et al., 2017), arcyriaflavin (Mervyn Bibb, unpublished data), gaburedin (Sidda et al., 2013), and venezuelin (Goto et al., 2010) (Table 1). Similar results were obtained by Kim et al. (Kim et al., 2020) in their antiSMASH analysis of the genome sequence of *S. venezuelae* ATCC 10712 (see later for further discussion). We also identified 1.5 kb terminal inverted repeats at the ends of the chromosome similar to those found in other *Streptomyces* linear replicons (Tidjani et al., 2020), and a potential telomere structure (see details in Supplementary material).

**Table 1:** The 34 Natural Product BGCs Identified After Manual Curation of an AntiSMASH v 5.0 (Blin et al., 2019) Analysis of the NRRL B-65442 Chromosome Sequence. The Seven Previously Identified NRRL B-65442 BGCs With Their Characterised Products Are Indicated by Asterisks. While Not Derived From NRRL B-65442, the Products of BGC 6 ((+)-isodauc-8-en-11-ol; (Rabe et al., 2015)) and BGC 29 (Foroxymithine; (Kodani et al., 2015)) Were Determined from Experiments With the ATCC 10712 Strain.

BGC	Type	From nt	To nt	Known or most similar BGC
1	Ectoine	237,110	247,526	Ectoine
2	Terpene	273,798	294,749	Geosmin
3	Type 1 PKS—Type 3 PKS	519,875	549,437	Venemycin*
4	NRPS-like	554,017	582,919	Watasemycin*
5	Ripp (lanthipeptide)	613,767	629,532	
6	Terpene	633,034	634,179	(+)-isodauc-8-en-11-ol
7	Ripp (lanthipeptide)	716,117	728,969	Venezuelin*
8	Indole	871,837	882,258	Arcyriaflavin*
9	NRPS-like	1,038,563	1,060,601	Chloramphenicol*
10	tRNA-dependent cyclodipeptide synthase	2,070,178	2,090,903	
11	Siderophore	2,798,699	2,809,633	Desferrioxamine B
12	Ripp (lasso peptide)	3,410,820	3,433,179	Albusnodin
13	NRPS-like	4,408,114	4,450,353	
14	Other	4,520,077	4,530,222	Gaburedins*
15	Melanin	5,001,915	5,010,131	
16	Other	5,475,407	5,516,513	
17	Ripp (thiopeptide)	5,525,842	5,558,923	
18	Type 3 PKS	5,784,219	5,822,500	Flaviolin
19	Siderophore	5,872,827	5,885,289	
20	Siderophore	5,938,343	5,952,704	
21	Type 2 PKS	6,473,487	6,545,282	Jadomycin*
22	NRPS-like	6,673,724	6,714,283	
23	NRPS-PKS	6,719,994	6,853,949	
24	Terpene	7,020,609	7,045,713	Hopene
25	Ripp (Lanthipeptide)	7,061,572	7,084,199	SapB
26	Bacteriocin	7,127,295	7,138,149	Linocin M18
27	Type 2 PKS	7,403,744	7,476,256	Spore pigment
28	Melanin	7,482,101	7,492,490	Melanin
29	NRPS	7,704,299	7,757,163	Salinichelins
30	Terpene	7,785,725	7,805,848	2-methylisoborneol
31	Type 3 PKS	7,943,145	7,984,236	Alkylresorcinol
32	Insecticidal protein	8,173,009	8,194,753	
33	Terpene	8,196,208	8,197,119	
34	NRPS	8,208,224	8,212,381	

### Curing of pSVJI1

To our knowledge, this is the first time that this plasmid has been observed in *S. venezuelae*. To verify that this was indeed an extra-chromosomal replicon, we set out to cure this putative plasmid by targeted deletion of its *parB*, which encodes a key component of the essential plasmid partitioning system (Funnell, 2016), using CRISPR/Cas9-based genome editing. Candidate spacer sequences were identified with CRISPy-web (Blin et al., 2016) and possible off-target sequences identified by blastN searches against the full-genome assembly. The chosen spacer sequence (5′-AGGAGGCAGAGTTCCTGCAA-3′) was then cloned in the pCRISPomyces-2 vector (Cobb et al., 2015) and the resulting plasmid pIJ13001 was transferred to *S. venezuelae* NRRL B-65442 by conjugation from *E. coli*; hundreds of putative apramycin-resistant exconjugants were obtained that yielded very small colonies, most of which failed to grow when re-streaked on selective medium, suggesting rapid loss of the vector. After subsequent growth on non-selective medium, three of the 16 apramycin sensitive exconjugants that were tested for the presence of the 158 kb plasmid failed to yield PCR products, indicating loss of the replicon (Supplementary Fig. S3).

To further verify plasmid loss, two of the exconjugants were subjected to whole-genome Illumina sequencing (together with the parental *S. venezuelae* NRRL B-65442 as a positive control) by MicrobesNG (Birmingham, UK). The trimmed Illumina readings were mapped to the full-genome assembly (CP018074.1 and CP018075.1). While the whole-genome data for the parental strain provided even coverage across both chromosome and plasmid sequences (with mean coverages of 76× and 60×, respectively), the whole-genome data from the two plasmid-cured candidates mapped exclusively to the chromosome with mean coverages of 90× and 332× (Supplementary Fig. S5).

The Illumina readings and contigs generated during this work (Supplementary Table S4) have been submitted to NCBI (BioProject Accession: PRJNA638164; see Supplementary Table S1 for full details); the original MicrobesNG data is available at https://microbesng.com/portal/projects/ED00339B-B15D-7943-891E-7045CACFA2D5/

Loss of the plasmid, which we named pSVJI1 (‘plasmid Streptomyces Venezuelae John Innes 1’), had no obvious phenotypic consequences. All three of the plasmid-cured derivatives, named *S. venezuelae* SS-292, that were examined developed lawns of green spores and spore chains (examined by cover slip impressions) that were indistinguishable from the parental strain *S. venezuelae* NRRL B-65442 (see Supplementary Fig. S4).

### Comparison of the Genome Sequence of *S. venezuelae* NRRL B-65442 With that of ATCC 10712

In addition to obtaining Illumina whole-genome sequences of NRRL B-65442, SS-292-1 and SS-292-2, we also sequenced the 10712-type strain acquired directly from ATCC (Supplementary Table S4). The MicrobesNG data yielded a very fragmented assembly (over a thousand contigs with N50 just over 10 kb; see the MicrobesNG project link above) but did confirm the presence of the 158 kb plasmid (see Supplementary Table S5 and Fig. S5).

While we were writing this manuscript, a high-quality genome sequence of the type strain ATCC 10712 (accession CP029197.1, length 8,223,505 bp) was published (Kim et al., 2020). Surprisingly, given the results of our Illumina sequencing, this sequence does not include the 158 kb plasmid. Comparison of this submission with our new genome sequence (CP018074.1, length 8,222,198 bp) using dnadiff from the MUMmer suite of programs (Version 4; Marçais et al., 2018) revealed only 46 nucleotide mismatches and 34 nucleotide deletions or insertions, equivalent to 99.99903% nucleotide sequence identity; this is higher than the expected accuracy of any DNA sequencing technology (Pfeiffer et al., 2018; Salk et al., 2018; Ma et al., 2019) and thus these differences may simply reflect sequencing errors. This high level of identity contrasts with the much lower level obtained when performing the same megablast alignment of NRRL B-65442 (CP018074.1; the JIC strain) with the recently published genome sequence for *S. venezuelae* ATCC 10595, the “Illinois strain” (CP029195.1; Kim et al., 2020); overall identity was 98.78% with only 90% of coverage of the NRRL B-65442 chromosome by the ATCC 10595 chromosome. Thus, despite the difference in spore pigmentation, NRRL B-65442 is clearly very closely related to the ATCC type strain 10712.

### Identification of a Single Nucleotide Substitution that Results in a Change in Spore Pigmentation

**Fig. 2:**
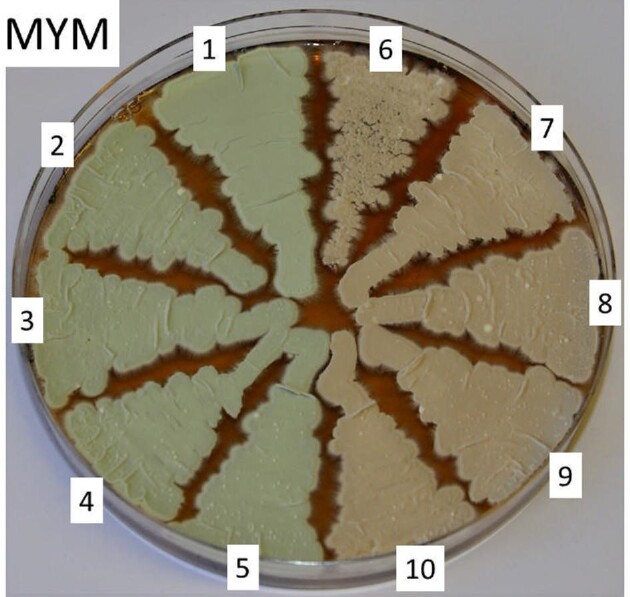
Sporulation phenotypes: 1, *S. venezuelae* NRRL B-65442; 6, *S. venezuelae* ATCC 10712; 2–5, *S. venezuelae* ATCC 10712 exconjugants with the *vnz_33525* allele from NRRL B-65442; 7–10, *S. venezuelae* ATCC 10712 exconjugants that had retained the parental *vnz_33525* allele. The strains were grown on MYM agar for six days at 30°C. The original RAW file is available from the authors upon request.

Analysis of the 80 single nucleotide differences between the NRRL B-65442 and ATCC 10712 genome sequences revealed that a C at nucleotide position 7444557 of the NRRL B-65442 sequence was replaced by a T in the ATCC 10712 genome sequence in *vnz_33525*. This resulted in the substitution of an arginine in NRRL B-65442 by a tryptophan in ATCC 10712 in a protein that encodes a FAD-dependent monooxygenase involved in spore pigment biosynthesis (it is a homologue of *whiE*_ORF VIII of *Streptomyces coelicolor*) (Kelemen et al., 1998; Yu & Hopwood, 1995). Inspection of the 97 most similar homologues of Vnz_33525 after blastP (https://blast.ncbi.nlm.nih.gov/Blast.cgi) analysis revealed that all possessed a tryptophan at the position of sequence divergence. To assess whether this nucleotide discrepancy was responsible for the difference in spore pigmentation, we carried out an allele replacement in ATCC 10712 using cosmid PI2_G12 (made from NRRL B-65442 genomic DNA; Bibb et al., 2012, http://strepdb.streptomyces.org.uk/cgi-bin/cosmids.pl?accession=CP018074&width=900) that contained an insert with just this single nucleotide difference. Replacement of the *vnz_33525* allele of ATCC 10712 with that of NRRL B-65442 (see Supplementary material) resulted in green spores (Fig. 2), thus explaining the discrepancy in spore pigmentation. Presumably, this mutation occurred at some point in the subculturing of the original ATCC 10712 strain giving rise to NRRL B-65442.

## Discussion

*S. venezuelae* NRRL B-65442 is an excellent model strain for the analysis of the control of development in filamentous actinobacteria. The adoption of this species has revealed a new understanding of the regulatory networks that direct morphological development in streptomycetes (Al-Bassam et al., 2014; Tschowri et al., 2014; Bush et al., 2013, 2015, 2016, 2019; Gallagher et al., 2020; Haist et al., 2020) and allowed cell biological analysis across the complete life cycle using fluorescence time-lapse imaging (Donczew et al., 2016; Schlimpert et al., 2016, 2017; Fröjd & Flärdh, 2019). Analysis of its genome sequence has also led to the identification of novel specialised metabolites (Goto et al., 2010; Sidda et al., 2013; Thanapipatsiri et al., 2016; Inahashi et al., 2017) and new insights into antibiotic biosynthesis (Fernández-Martínez et al., 2014).

The results described here demonstrate that our model strain, now designated NRRL B-65442, which was obtained from the Vining laboratory, is very closely related to the ATCC type strain 10712. Moreover, we were able to identify a single nucleotide substitution in *vnz_33525* that is responsible for the difference in spore pigmentation of the two strains. Streptomycete spore pigments are derived from type II polyketide biosynthetic gene clusters that likely produce cyclised or aromatic polyketides that are modified by tailoring enzymes encoded by the cluster (Davis & Chater, 1990). The FAD-dependent monooxygenase encoded by *vnz_33525* is one such tailoring enzyme. Homologues of this enzyme are involved in spore pigment biosynthesis in *S. coelicolor* A3(2), where its absence led to the production of spores with a greenish tinge rather than the usual grey colour (Kelemen et al., 1998; Yu & Hopwood, 1995), and in *Streptomyces halstedii*, where its absence resulted in lilac rather than green spores (Blanco et al., 1993). The precise nature of the oxidative modification (e.g., hydroxylation) carried out by Vnz_33525 and why it should result in a change in colour remain to be resolved.

Our studies also revealed the presence of a 158 kb plasmid, pSVJI1, in NRRL B-65442 that was not reported in the recent ATCC 10712 genome sequence (Kim et al., 2020) (although it was evident in our Illumina sequencing of the same ATCC strain). Interestingly, a blast search using the full 158 kb plasmid sequence identified a recently deposited whole-genome shotgun assembly from strain *S. venezuelae* O-10.2 (NCBI bio-project accession PRJNA441708); the plasmid is almost fully covered (98%) by three contigs with nearly 100% identity; the genome assembly was performed by the DOE Joint Genome Institute, GOLD Project ID Gp0224262 and the origin of the strain is unknown.

We have also reported the application of CRISPR-Cas9 technology to cure pSVJI1, an approach that should be broadly applicable in future studies of plasmid biology in streptomycetes and that has also been used in parallel with this study for plasmid curing in *Streptomyces clavuligerus* (Gomez-Escribano et al., submitted for publication).

## Supplementary Material

kuab035_Supplemental_File
