# Supplementary material for: Streptomyces venezuelae NRRL B-65442: genome sequence of a model strain used to study morphological differentiation in filamentous actinobacteria
Source: J Ind Microbiol Biotechnol. 2021 Jun 8;48(9-10):kuab035. doi: 10.1093/jimb/kuab035 (PMC8788739; doi:10.1093/jimb/kuab035)
Supplement: kuab035_Supplemental_File [file kuab035_supplemental_file.pdf]

Supplementary data for "*Streptomyces venezuelae* NRRL B-65442: genome sequence of a model strain used to study morphological differentiation in filamentous actinobacteria" Juan Pablo Gomez-Escribano, Neil A. Holmes, Susan Schlimpert, Maureen J. Bibb, Govind Chandra, Barrie Wilkinson, Mark J. Buttner, Mervyn J. Bibb

Table of Contents

Access to the sequence data reported in this work (Table S1) 2

MATERIALS AND METHODS 2

Strains, culture conditions and allele replacement 2

History of *S. venezuelae* strains 3

Table S2. *S. venezuelae* strains used in this study. 4

PHENOTYPIC CHARACTERISATION OF STRAINS 5

The sporulation phenotype of the JIC model strain is different to that of the type strain 5

Assessment of chloramphenicol production 5

Figure S1. Phenotype of spore lawns of the different *S. venezuelae* strains 6

Figure S2. LC-MS analysis of chloramphenicol production 7

GENOME SEQUENCING. 9

PacBio sequencing 10

Illumina sequencing 10

Chromosome walking by PCR and Sanger sequencing for extension of the PacBio chromosome 10

Analysis of sequence data 11

Table S3. Summary of the output for each PacBio genome assembly project. 11

Table S4a. Summary of the trimmed reads output for each Illumina genome assembly project. 12

Table S4b. Summary of the assembled contigs output for each Illumina genome assembly project. 12

CURING OF THE PLASMID pSVJI1 12

Use of CRISPR-cas9 for plasmid curing 12

PCR test of plasmid-cured candidate clones 12

Figure S3. Conjugation and replication plates. 13

Phenotypic analysis of pSVJI1 cured strains 14

Figure S4. Analysis of sporulation by plasmid-cured strains. 14

Assessment of plasmid-cured clones by whole-genome sequencing 14

Figure S5. Qualimap analysis of Illumina data. 15

Table S5. Coverage of each replicon by Illumina reads 17

Extension of the end of the replicons and identification of putative terminal inverted repeats and telomere 17

Sequences extending the ends of the 2016 chromosome and plasmid 18

REFERENCES 19

Access to the sequence data reported in this work (Table S1)

Table S1. The Whole Genome Shotgun projects described in this paper have been deposited at DDBJ/ENA/GenBank through the NCBI Submission Portal. The Illumina trimmed reads have been deposited at NCBI's Sequence Read Archive (SRA). This table provides the relevant information to access the DNA sequences and other specific details of the projects.

<table>
<tr><td colspan="5">Streptomyces venezuelae NRRL B-65442- High-quality genome assembly, made public in Nov 2016
BioProject Accession: PRJNA353366; BioSample Accession: SAMN06014621
Assembly Accession: GCA_001886595
The version described in this paper is version 1 (i.e. CP018074.1)</td></tr>
<tr><td>Accession</td><td>Replicon</td><td colspan="2">Sequence version deposited</td><td>Technology</td></tr>
<tr><td>CP018074</td><td>Chromosome</td><td colspan="2">Chromosome_2016-09-01</td><td>PacBio+Sanger</td></tr>
<tr><td>CP018075</td><td>Plasmid</td><td colspan="2">plasmid_2016-09-01</td><td>PacBio+Sanger</td></tr>
<tr><td colspan="5">Streptomyces venezuelae NRRL B-65442 plasmid-cured derivatives, parental control, and type deposit
BioProject Accession: PRJNA638164
The version described in this paper is version 1 (i.e. XXXX01000000)
MicrobesNG project site: https://microbesng.com/portal/projects/ED00339B-B15D-7943-891E-7045CACFA2D5/</td></tr>
<tr><td>BioSample Accession</td><td>Genome (contigs) Accession</td><td>Strain (clone)</td><td>MicrobesNG reference (barcode)</td><td>Sequence Read Archive (SRA)</td></tr>
<tr><td>SAMN15180651</td><td>JABVZL000000000</td><td>SS-292-1</td><td>13400</td><td>SRR11960413</td></tr>
<tr><td>SAMN15180652</td><td>JABVZM000000000</td><td>SS-292-2</td><td>13401</td><td>SRR11960412</td></tr>
<tr><td>SAMN15180653</td><td>JABVZN000000000</td><td>NRRL B-65442</td><td>13398</td><td>SRR11960411</td></tr>
<tr><td>SAMN15180654</td><td>JABVZO000000000</td><td>ATCC 10712</td><td>13399</td><td>SRR11960410</td></tr>
</table>

MATERIALS AND METHODS

Strains, culture conditions and allele replacement

S. venezuelae NRRL B-65442 is the model strain used at the John Innes Centre (JIC). *S. venezuelae* ATCC 10712, ATCC 10595, NRRL 2277, and NRRL B-902 were obtained directly from the ATCC or NRRL culture collections, respectively. *S. venezuelae* "strain 13s" and "Sven_ Dalhousie" were kindly provided by Prof. David Jakeman (Dalhousie University, Canada). *S. venezuelae* strains were cultivated in liquid or agar MYM [1], Difco Nutrient Agar (DNA; Becton Dickinson 213000), TSB:YEME [2] 50:50, and SFM(MS) [2] culture media. The antibiotics chloramphenicol, apramycin, kanamycin, and nalidixic acid were purchased from Sigma. *Escherichia coli* DH5α was used as general purpose cloning host following established procedures [3]. *E. coli* ET12567/pUZ8002 was used as donor strain in the *E. coli-Streptomyces* conjugations following established methods [2].

For allele replacement, cosmid Pl2_G12 (made from NRRL B-65442 genomic DNA; Bibb et al., 2012; http://strepdb.streptomyces.org.uk/cgi-bin/cosmids.pl?accession=CP018074&width=900) was modified by PCR-targeting to replace the ampicillin resistance gene with the apramycin/*oriT* cassette from pIJ773 [4] using the following oligonucleotides as PCR primers;

AmpApr2F, aaatgaagttttaaatcaatctaaagtatatatgagtaacggaataggaacttatgagc; AmpApr2R, ataatggtttcttagacgtcaggtggcacttttcggggaacttcgaagttcccgccag

The modified Pl2_G12 cosmid was introduced into *E.coli* ET12567/pUZ8002 by transformation and from there into *S. venezuelae* ATCC 10712 by conjugation. After subculturing one of the exconjugants in the absence of selection, we were able to identify apramycin sensitive colonies. Four apramycin sensitive green isolates and four apramycin sensitive grey isolates were selected for further analysis. For each of these eight strains, we then PCR-amplified and sequenced *vnz_33525* to confirm its origin (i.e., derived from either NRRL B-65442 or ATCC 10712). We also PCR amplified and sequenced *vnz_18620* (a homologue of *sigF* of *Streptomyces coelicolor*A3(2)), *vnz_16900* (a homologue of *dnaK* of *S. coelicolor* A3(2)), and *vnz_11145* (a homologue of *nirB* of *S. coelicolor* A3(2)). Each of these latter three genes also exhibited single nucleotide differences when the two genome sequences were compared (for the *sigF* homologue at chromosomal nucleotide positions 4111917 (T) in NRRL B-65442 and 4112465 (G) in ATCC 10712, for the *dnaK* homologue at chromosomal nucleotide positions 3752628 (A) in NRRL B-65442 and 3753177 (G) in ATCC 10712, and for the *nirB* homologue at chromosomal nucleotide positions 2461679 (A) in NRRL B-65442 and 2462229 (C) in ATCC 10712. For each of the four green isolates, all four sequences were consistent with introduction of the *vnz_33525* allele from NRRL B-65442 into the ATCC 10712 chromosome, thus confirming allele exchange; conversely, in the grey isolates all four sequences corresponded to the ATCC 10712 genome sequence. The following oligonucleotides were used as PCR primers and for subsequent sequencing of the four genes: *vnz33525*: vnz33525_seqF1 ggaagaagggcggcatcc and vnz33525_seqR1 gcaaggaagcggtcagtc; *vnz_18620*: sigF_seqF1 tgaccgtagttgacccagtc and sigF_seqR1 agcggtcgtcaggtcctc; *vnz_16900*:dnaK_seqF1 acctgggcacgactaactc and dnaK_seqR1 gagggacacgtcgaaggt; *vnz_11145*: nirB_seqF1 tctacacgaagatcaccggc and nirB_seqR1 accaccacgtccttgagg.

History of *S. venezuelae* strains

S. venezuelae was first described as a producer of chloromycetin [4] (later called chloramphenicol [5]) isolated from a soil sample from Caracas, Venezuela, hence the species name [6]. This first isolate, which received the strain codes Burkholder No. A65 by the original discoverers at Yale University, USA [6] and later No. 04745 by the Culture Bureau of Parke, Davis and Company at Detroit, USA [6], the company that originally exploited the production of chloramphenicol for clinical use, was deposited at ATCC as the type strain with number 10712 [7]. Concomitantly, a group at the University of Illinois (USA) also isolated a chloramphenicol producing strain from composted soil at the Illinois Agricultural Experiment Station at Urbana, USA; this strain was given the identifier “Gottlieb no. 8-44” [8] (also written as “DG 8-44”) and later deposited at ARS-NRRL as strain B-902.

The seminal work on *S. venezuelae* biology, particularly in relation to phage biology, was undertaken by Colin Stuttard’s group [9], while most of the work on the chemistry, biochemistry and genetics of chloramphenicol production by *S. venezuelae* was undertaken by Leonard C. Vining’s group [10], both at Dalhousie University in Halifax, Canada. Initial investigations were performed on a third isolate of *S. venezuelae* from the Upjohn Culture Collection (Upjohn Company, Kalamazoo, Michigan, USA), strain number UC2374 [11], also identified in Stuttard’s and Vining’s publications as *Streptomyces* sp. 3022a (they also deposited the strain in the Prairie Regional Laboratory culture collection (Saskatoon, Canada) as PRL 1951) [12]. Most of the initial work on chloramphenicol by Vining’s group was undertaken with a spontaneous mutant of this strain called “strain 13s” which they selected for increased production of the antibiotic [11, 13–15] (and robust sporulation, David Jakeman, personal communication). *Streptomyces* sp. 3022a, and therefore also 13s, were later identified as *S. venezuelae* [11, 16]. Both Stuttard and Vining also worked with the type strain, obtained as ISP5230 first from Kaken Chemical Co., Tokyo, Japan (as strain KCC

S-0526) [11] and as ATCC 10712 (ISP5230) from Elizabeth Wellington (then at the University of Liverpool, UK) [10, 16]. This complex history is summarised in Table S2.

Table S2. *S. venezuelae* strains used in this study.

Strain designations	Remarks	Refs.
ATCC 10712<br>NRRL 2277<br>P. Burkholder A-65<br>Parke Davis & Co. PD 04745	Type strain of *S. venezuelae* Ehrlich *et al*. 1948. First isolate, from soil from Caracas (Venezuela). Original deposit directly from Parke Davis & Co.	[4, 6, 7]
ISP 5230	Type strain of *S. venezuelae* Ehrlich *et al*. 1948. Uncertain deposit path; NRRL state: "Shirling, ISP from L. Anderson"; unsure whether it originated directly from Parke Davis & Co.	
ATCC 10595<br>NRRL B-902<br>DB 8-44	Isolate of *S. venezuelae* from Illinois soil by David Gottlieb.	[8]
Upjohn UC2374<br>3022<br>3022a<br>PRL 1951	Upjohn Company (Kalamazoo, Michigan, USA). Also known as *Streptomyces* sp. 3022 = 3022a and deposited in the Prairie Regional Laboratory culture collection (Saskatoon, Canada) as PRL 1951. The first publication [12] mentioning this strain states UC2376 as the strain number for 3022a but this is most likely an error, since UC2376 corresponds to *Streptomyces lincolnensis* ATCC 25466, and the later publication describes 3022a as UC2374 [11].	[12, 11, 17]
13s	Chloramphenicol overproducer, spontaneous mutant of UC2374 =3022a=PRL1951; kindly provided by David Jakeman (University of Dalhousie, Canada).	[12, 15, 18]
Sven_Dalhousie	Working strain of Prof. Leo Vining's group, kindly provided by David Jakeman (University of Dalhousie, Canada). Referred to in the Vining's publications as ATCC 10712 and ISP 5230.	[19]
NRRL B-65442	John Innes Centre isolate established as a model strain.	This work
SS292	NRRL B-65442 cured of pSVJI1	This work

PHENOTYPIC CHARACTERISATION OF STRAINS

The sporulation phenotype of the JIC model strain is different to that of the type strain

All of the work carried out at the JIC, and by others who have received our strain, has been performed on a *S. venezuelae* strain obtained by Mervyn Bibb as a gift from Diversa Corp. (San Diego, USA) and described as the type strain ATCC 10712; Diversa also provided a genome sequence for this isolate [20]. Recently, during an independent project, we sourced a new stock of the type strain ATCC 10712 directly from ATCC; strikingly the newly acquired stock produced a lawn of grey-pigmented spores when cultivated on MYM agar medium in contrast to the green-pigmented spores produced by the JIC isolate; this prompted us to delve into the history of chloramphenicol producing *Streptomyces* species.

We obtained the type strain from both the ATCC (no. 10712) and NRRL (no. 2277) culture collections, as well as Gottlieb's isolate 8-44 (also from both culture collections as ATCC 10595 and NRRL B-902). All of them produced grey-pigmented lawns of spores when cultivated on MYM agar medium, contrasting with the green-pigmented spores produced by our strain (Figure S1). Given that Diversa Corp. had received the strain from the Vining laboratory, we then acquired two additional strains (kindly provided by Prof. David Jakeman (Dalhousie University, Canada)) that had been used by the Canadian group: the chloramphenicol overproducer "strain 13s" and a strain thought to be the type deposit ISP 5230 (supposedly synonymous with ATCC 10712) which we referred to as "Sven_ Dalhousie"; only strain Sven_ Dalhousie produced a lawn of green spores on MYM agar medium, while "strain13s" produced a grey lawn of spores similar to the other strains (Figure S1). On SFM agar medium most of the strains produced pale grey lawns of spores (Figure S1) probably attributable to relatively poor sporulation compared to MYM agar. In addition to these strains, we found a viable stock of the Upjohn strain UC2374 [17] (and therefore expected to be the strain used by Stuttard's and Vining's groups as *Streptomyces* sp. 3022a). This strain also developed a grey lawn of spores on MYM agar medium (data not shown). Thus, the only *S. venezuelae* strains to produce green spores were the JIC isolate and Sven_ Dalhousie. Scanning Electron Microscopy (SEM) of fully sporulated colonies from each of the strains shown in Figure S1 failed to reveal any discernible differences in morphology (data not shown).

Assessment of chloramphenicol production

We analysed chloramphenicol production by all of the strains shown in Figure S1 by HPLC-PDA-MS. Production by the JIC strain (NRRL B-65442) (1.5 mg/l) was not significantly different from most of the others (0.2-1.1 mg/l), while "strain 13s" gave 11.5 mg/l, in agreement with reports from the Vining group. Experimental details follow.

MYM liquid cultures were inoculated with spores and incubated with orbital shaking for 9 days. Chloramphenicol was extracted from 35 ml of supernatant with an equal volume of ethyl acetate; ethyl acetate was recovered and completely evaporated; the dried residue was resuspended in 1 ml of 50% methanol in water; 5 microliters were analysed by HPLC as previously described [21] coupled with MS detection in negative mode on a Shimadzu LC-MS system equipped with a NexeraX2 liquid chromatograph (LC30AD) fitted with a Prominence photodiode array detector (SPD-M20A) and an LCMS-IT-ToF mass spectrometer; samples were injected in a Kinetex XB C18 2.6-um, 100-Å, 50- by 2.10-mm column (part no. 00B-4496-AN; Phenomenex, USA). Commercially available chloramphenicol (Sigma C0378) was used as standard.

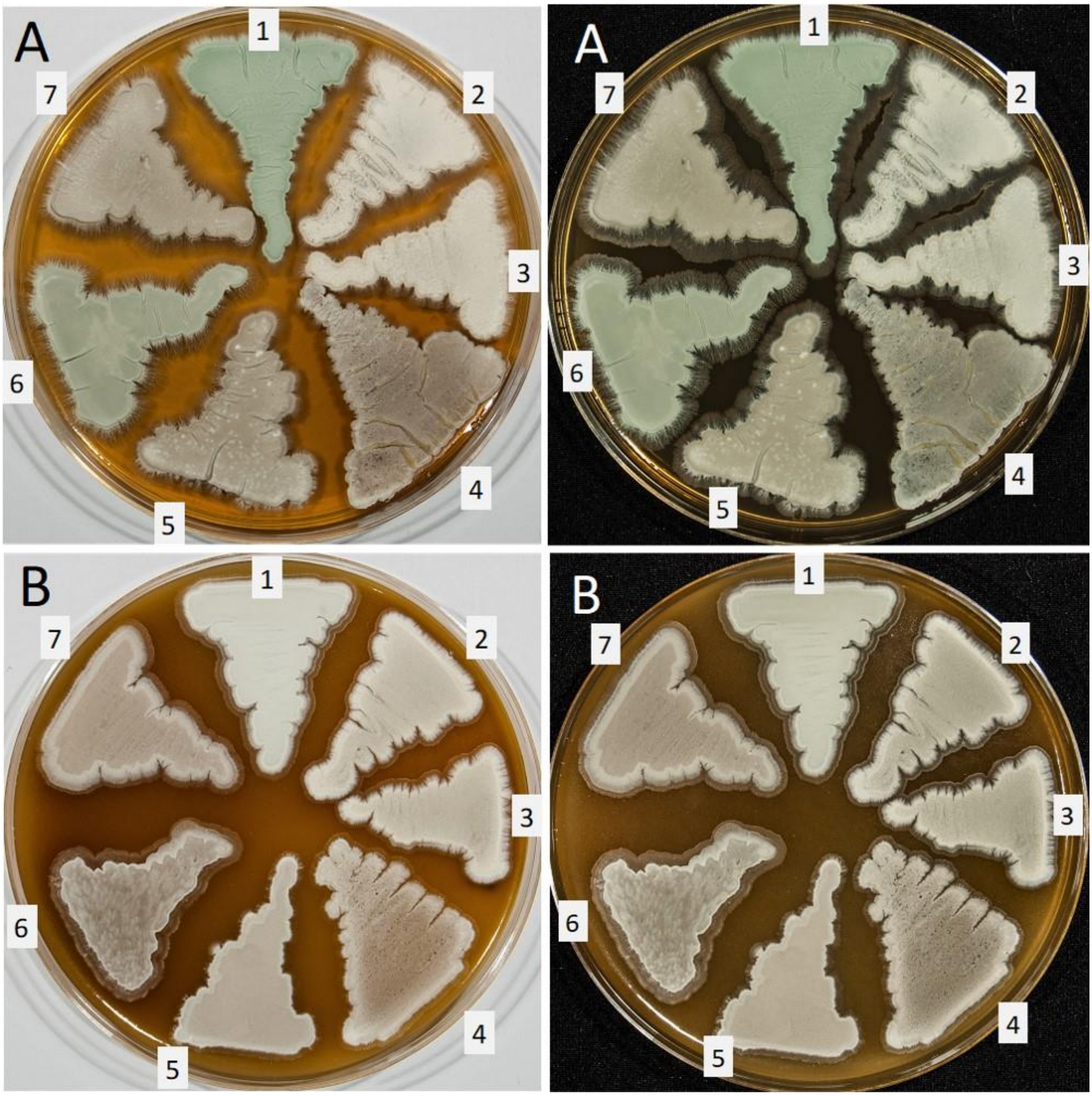

Figure S1. Phenotype of spore lawns of the different *S. venezuelae* strains

S. venezuelae strains were grown on MYM (A) and SFM (B) agar media for 7 days. 1, NRRL B-65442; 2, NRRL B-902; 3, ATCC 10595; 4, NRRL 2277; 5, ATCC 10712; 6, Sven_ Dalhousie; 7, strain 13s. The original RAW camera files are available from the authors upon request.

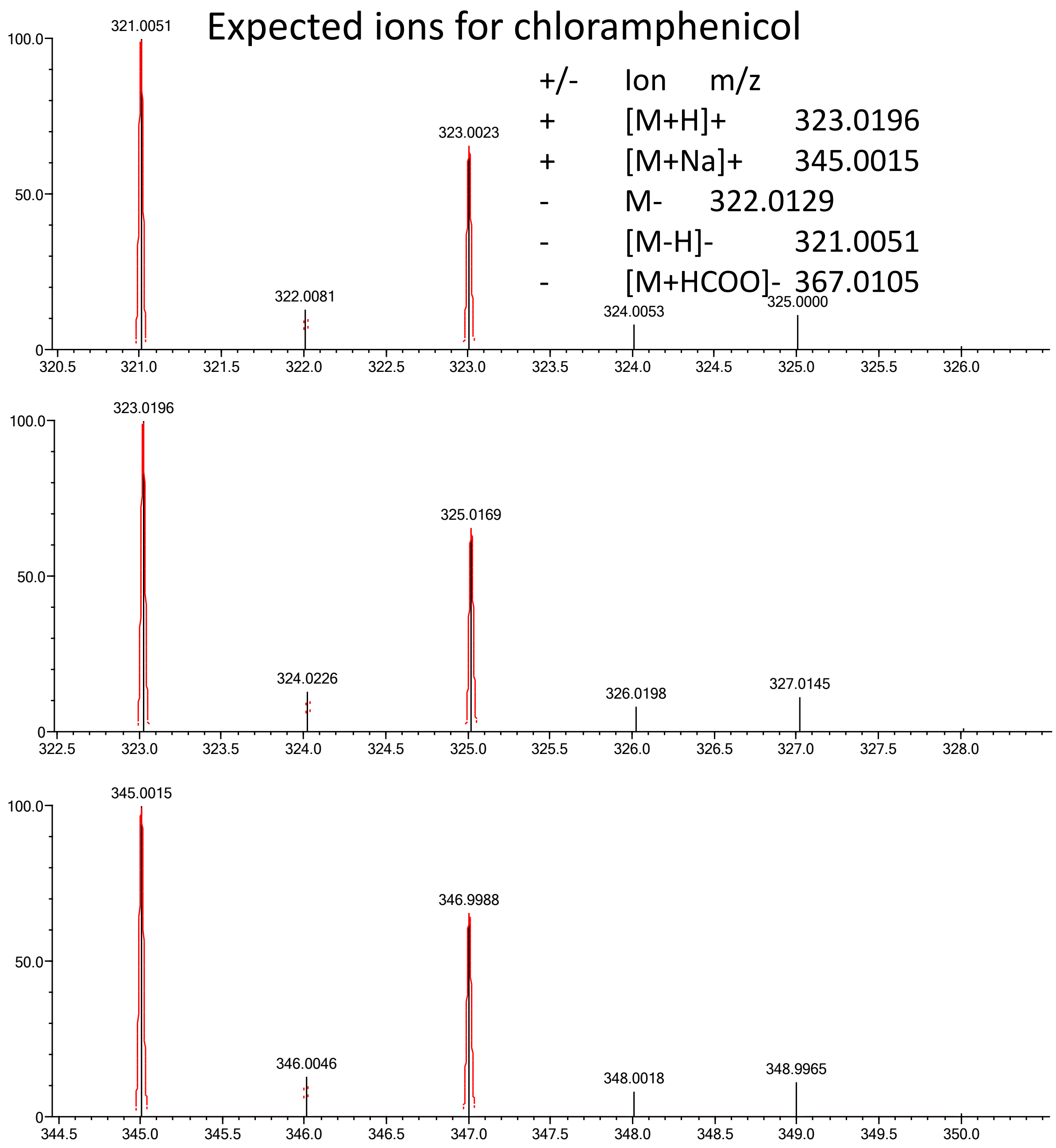

Figure S2. LC-MS analysis of chloramphenicol production

Simulation of spectra based on the molecular formula of chloramphenicol justifying the selection of 321.0056 m/z for the negative mode extracted ion chromatograms shown below.

While more qualitative than quantitative, these results demonstrate that all strains produce chloramphenicol in readily detectable amounts under these laboratory conditions.

A. Extracted Ion Chromatogram (EIC) of the negative-mode data for the expected $[M\text{-}H]^-$ ion of chloramphenicol, 321.0056 m/z; all samples contain chloramphenicol, with strain 13s showing the highest production. The strains obtained from the culture collections were grown from either single colony isolates or from mixtures of multiple colonies (to try to cater for the morphological heterogeneity observed when recovering the isolates from freeze-dried cultures).

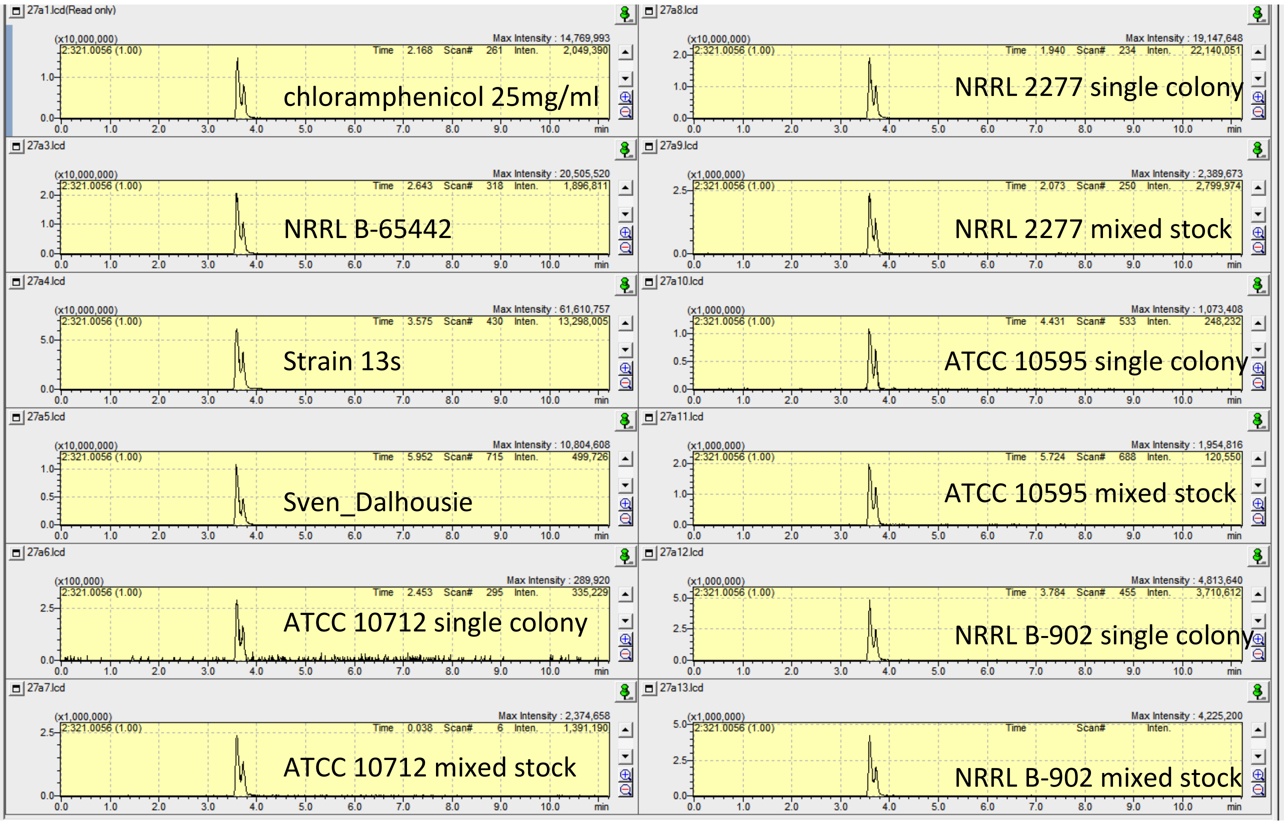

B. Production of chloramphenicol (cml) calculated from peak areas at 273 nm (as described previously [21])

**Strain**	**cml in broth ug/ml**
NRRL B-65442	1.48
Strain13s	11.45
Sven_Dalhousie	0.77
ATCC 10712 single colony	0.48
ATCC 10712 mixed stock	0.50
NRRL 2277 single colony	1.11
NRRL 2277 mixed stock	0.38
ATCC 10595 single colony	0.42
ATCC 10595 mixed stock	0.17
NRRL B-902 single colony	0.19
NRRL B-902 mixed stock	0.37

C. Chromatogram at 273 nm (see [21] for details) of representative samples. The peaks of chloramphenicol are readily identified in the samples with the highest amounts.

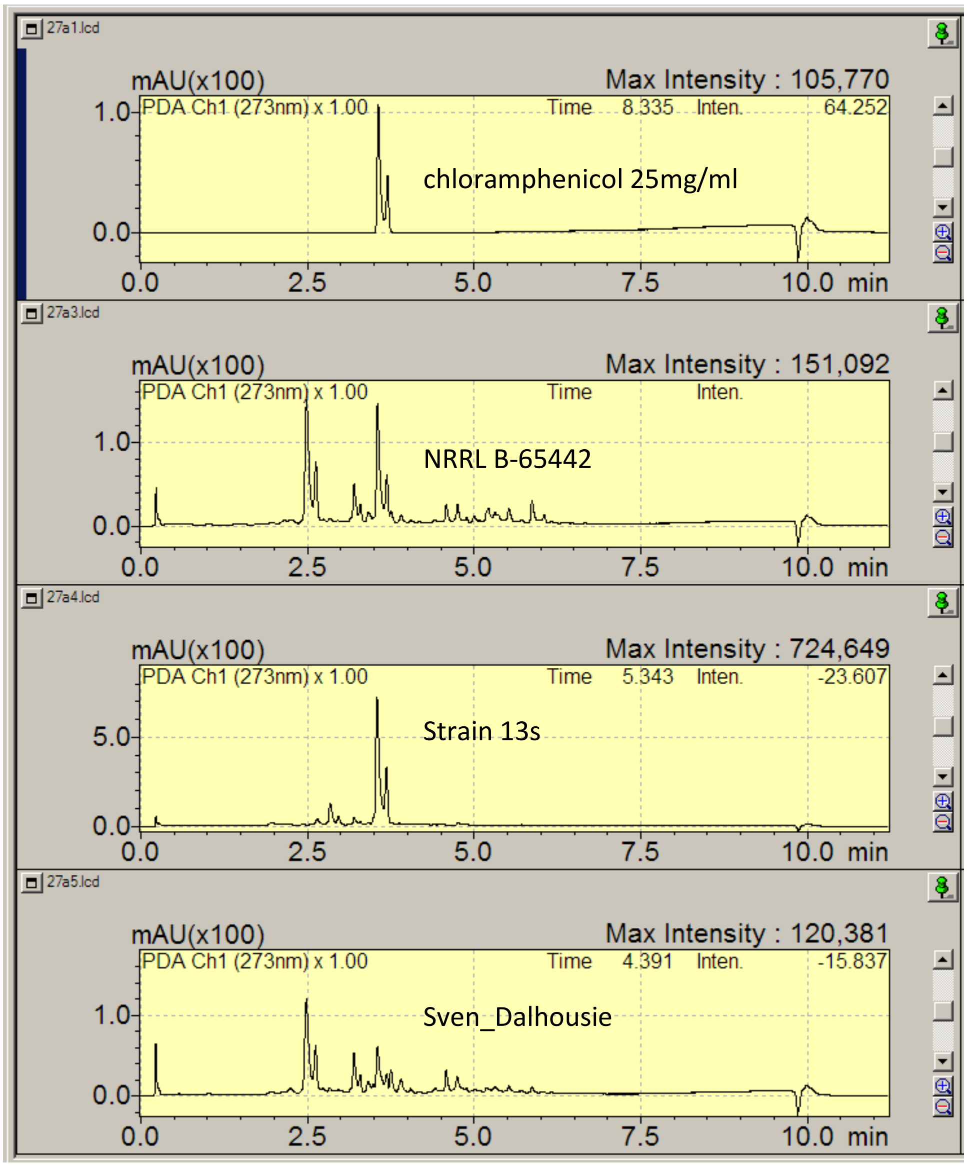

GENOME SEQUENCING.

High molecular weight DNA samples were extracted following the salting-out protocol [2] from early-stationary-phase cultures; 50 ml of TSB:YEME [2] 50:50 in a 250 ml Erlenmeyer flask containing a spring were inoculated with 5 µl of spore stock; the culture was incubated at 30 °C with shaking at 250 rpm for about 18 h; phase contrast microscopy confirmed that the mycelium was well grown, dispersed, with no indication of fragmentation or sporulation (this medium was preferred to MYM because it does not support sporulation in liquid, and yields dispersed mycelium without fragmentation that greatly facilitates the extraction of high-quality high-molecular weight genomic DNA). Sample quality was first assessed by standard agarose-gel electrophoresis and then pulse-field gel electrophoresis, and quantification was performed with NanoDrop (Thermo Fisher Scientific) and Qubit (Thermo Fisher Scientific).

PacBio sequencing

PacBio RSII SMRT sequencing (Pacific Biosciences of California, Inc) was commissioned to the Earlham Institute (Norwich Research Park, Norwich, NR4 7UZ, United Kingdom). Data was processed and assembled with HGAP.3 (Table S3) using the SMRTpipe program with the following parameters (as provided by the supplier):

minlongreadlength : 6000	ovlminlen : 40	splitbestn : 10
mincorcov : 6	genomesize : 9000000 (9 Mb)	totalbestn : 24
maxdivergence : 30	computelengthcutoff : True	targetchunks : 6
mersize : 14	ovlerrorrate : 0.06	readscore : 0.8
maxslotperc : 1	minsubreadlength : 500	placerepeatsrandomly : True
xcoverage : 25	minlength : 100	
defaultfrgminlen : 500	enablemapqvfilter : True	

blasropts : -noSplitSubreads -minReadLength 200 -maxScore 1000 -maxLCPLength 16 minanchorsize : 12
sambam : True gff2bed : True pbalign_opts : --seed=1 --minAccuracy=0.75 --minLength=50 --algorithmOptions="-useQuality

Illumina sequencing

Illumina sequencing was commissioned to MicrobesNG (IMI - School of Biosciences, University of Birmingham, Edgbaston, Birmingham, B15 2TT, United Kingdom). The project was undertaken between 22nd February and 28th March 2018. Briefly, as extracted from the full protocol accessed at https://microbesng.uk/documents/5/MicrobesNG_Methods_Document_-_PDF.pdf on 5th May 2019: PCR-based libraries were prepared using a Nextera XT Library Prep Kit (Illumina, San Diego, USA) and sequenced on an Illumina HiSeq 2500 using a 250 bp paired-end protocol. Reads were adapter trimmed using Trimmomatic 0.30 with a sliding window quality cut-off of Q15. *De novo* assembly was performed on samples using SPAdes version 3.7, and contigs were annotated using Prokka 1.11. Tables S4a and S4b provide information about the output and quality of assemblies.

Chromosome walking by PCR and Sanger sequencing for extension of the PacBio chromosome

The ends of the chromosomal contig generated by PacBio assembly were extended in an attempt to cover the previously determined sequence (FR845719) by PCR amplification and Sanger sequencing of PCR products. PCR primers were designed using the published sequence FR845719 and the extended end of the contig. The oligonucleotides used as primers used were:

sven1, gaacgcgaggagaagaacag; sven2, tcctgcgaacgagtcctact; sven3, tacgacaccgactcatccac; sven4, cgaagtcccactggaagc; sven5, cccgaagaactacaccgaga; sven6, tcccaagatccactttcgac; sven7, gtgaccgtttcagagcgttt; sven8, ggtgtactcgagggctgaac; sven9, tcttggtttcgcgggtatt; sven10, gcatggagctccgtaaagac; sven11, gaatcctcagcagccacttc; sven12, caggtcatgaaccgacttcc; sven13, gtggcaggatgaccctatgt; sven14, cgaagtgccgtcaaagagtt; sven15, cttgacggtgggtcctctta; sven16, aggcatgggattcagttcag; sven17, acgactacggcacgctctat; sven18, ggtagctgtacgcgaaggag; sven19, agatcgtcacgagcgtcttc; sven20, accgcctgtatgtccgtact; sven21, cagaactcgaaccccaggta; sven22, acgcagactcgatacgttcc; sven23, ggtggcctgtgttcctttc; sven24, gctggtgcttctggtggt

Analysis of sequence data

General visualisation, analysis, and manipulation of DNA sequence data were performed with computer programs ApE (M. Wayne Davis, https://jorgensen.biology.utah.edu/wayned/ape/), Artemis [22], Artemis Comparison Tool [23], and NotePad++ (http://notepad-plus-plus.org/); mapping of next-generation sequencing reads and contigs was performed with BWA [24, 25] and SAMtools [26] as previously described; assembly files were visualised with BAMView [27] and their quality was assessed with Qualimap 2.2.1 [28, 29]; alignments and assembly of sequences was performed with the Staden Package [30, 31]. BLAST+ [32] searches were performed at the NCBI web server (http://www.ncbi.nlm.nih.gov/blast/), or on a standalone computer with prfectBLAST 2.0 [33]. Annotation of gene function and genetic features was performed with RAST [34, 35] and antiSMASH [36].

Table S3. Summary of the output for each PacBio genome assembly project.

**Sequencing information**	**(HGAP mapping_statistics_report)**
Number of SMRT cells	6
Mapped Subread Bases	1354028668
Mapped Polymerase Read Length Max	39041
Number of Aligned Reads	185845
Mapped Polymerase Bases	1367987958
Mapped Polymerase Read Length 95%	22880
Mapped Polymerase Read Length	7361
Mapped Read Length of Insert	5468
Mapped Reads	185845
Mapped Subread Length	5589
Mapped Subread Accuracy	0.8579
Mapped N50	11337
Mapped Subreads	242274
Mapped Subread N50	8557
**Assembly output**	
Total number of contigs	2
Minimum contig length	144576
Maximum contig length	8208916
Mean length of total contigs	4176746
N50 contig length	8208916
N80 contig length	8208916
N90 contig length	8208916
Total bases	8353492
Mean coverage	154.9333
Contigs, as matching published replicons (original name, size in bp)	
unitig_0 (chromosome)	8208916
unitig_1 (plasmid)	144576

Table S4a. Summary of the trimmed reads output for each Illumina genome assembly project.

Sample id	Median insert size	Mean coverage	Mean coverage excluding 0s	Number of reads	Number of reads with insert size >300
NRRL B-65442	589	77.8696	77.8841	1497876	998443
ATCC 10712	633	69.473	69.4856	1319874	861881
SS-292 clone 5	399	91.8553	91.8721	1836271	1031166
SS-292 clone 8	537	333.78	33.838	6235793	4827948

Table S4b. Summary of the assembled contigs output for each Illumina genome assembly project.

**Sample id**	**NRRL B-65442**	**ATCC 10712**	**SS-292-1 (clone SS5)**	**SS-292-2 (clone SS8)**
**# contigs (>= 0 bp)**	1218	1380	1250	1145
**# contigs (>= 1000 bp)**	1006	1141	1023	851
**Total length (>= 0 bp)**	8215207	8165202	8122334	8220307
**Total length (>= 1000 bp)**	8083241	8017009	7987284	8068672
**# contigs**	1145	1291	1145	947
**Largest contig**	81764	73806	72045	72781
**Total length**	8185808	8127713	8079194	8138935
**GC (%)**	72.32	72.28	72.37	72.39
**N50**	12512	10710	12268	15225
**N75**	6483	5845	6528	8116
**L50**	184	218	191	154
**L75**	411	473	416	338
**# N's per 100 kbp**	0	0	0.01	0

CURING OF THE PLASMID pSVJI1

Use of CRISPR-cas9 for plasmid curing

pCRISPomyces-2, constructed by Huimin Zhao's laboratory [37], was kindly provided by Rebecca Lo and Matt Hutchings (JIC, Norwich, UK). pIJ13101 was constructed by cloning the annealed oligonucleotides JP301 (5'-acgcAGGAGGCAGAGTTCCTGCAA-3') and JP302 (5'-aaacTTGCAGGAACTCTGCCTCCT-3') into pCRISPomyces-2 following the protocol in [38]. pIJ13101 was transferred to *S. venezuelae* NRRL B-65442 by conjugation from *E. coli* ET12567/pUZ8002 following established methods [2] and using nalidixic acid and apramycin to select for *Streptomyces* exconjugants.

Plenty of exconjugants resulted but with colony sizes that were much smaller than those usually observed with this strain (Fig. S3A). Upon streaking to DNA agar supplemented with nalidixic acid and with or without apramycin, most exconjugants had lost resistance to apramycin (Fig. S3B). Several potentially cured clones that were apramycin-sensitive were selected for PCR testing for the presence of the plasmid.

PCR test of plasmid-cured candidate clones

The following oligonucleotides primer pairs were used for PCR amplification (from 5' to 3'):
671 (ATGCCGACTCGCGACACCATCG) / 672 (TTGAACTGGCGAAGTCACCGGAGC)
673 (TTTGGCGGTACCAGACGTGGAGG) / 674 (TCAGTCGCTCGCCGTCGCTTCG)
675 (TGCGGCACCCTGTTACGCACG) / 676 (CAGGTGACGCACGCCTGCTCG)
677 (GTTCTCGCCGGGACTGAACCTGC) / 678 (GGATCGCTTCGTCTGTCTGCTGC)

In total 16 plasmid-cured candidate clones were tested and three provided negative PCR reactions with all primer pairs.

A

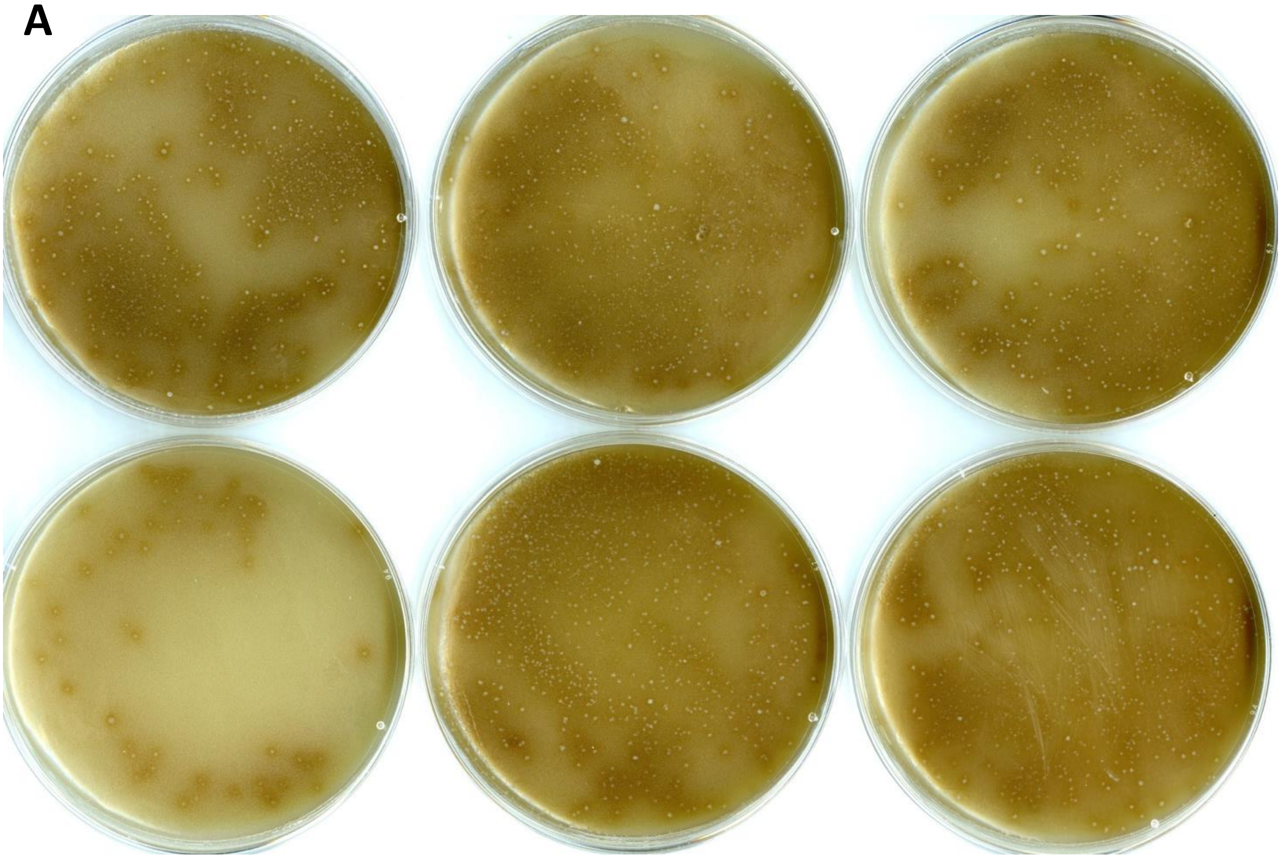

B

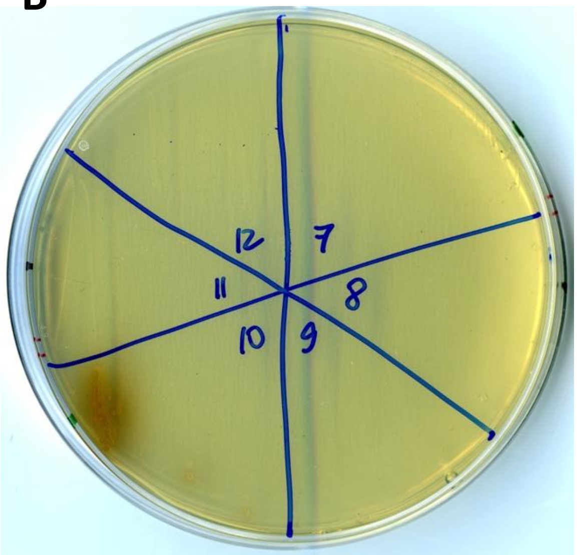

Figure S3. Conjugation and replication plates.

(A) Conjugation plates showing the appearance of small exconjugants. (B) Upon streaking to plates containing apramycin, most of the exconjugants had lost resistance to the antibiotic.

Phenotypic analysis of pSVJI1 cured strains

Microscopic analysis of cover slip impressions of colonies from three of the cured strains were performed as previously described [39]. In all cases, sporulation by the plasmid-cured derivatives was indistinguishable from that of a control exconjugant that had maintained the plasmid (Fig. S4) and to the parental strain (not shown).

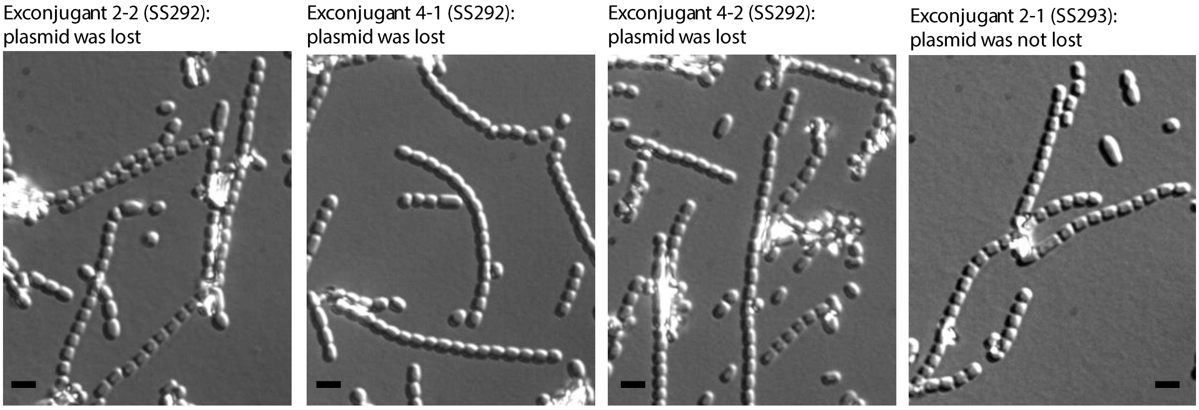

Figure S4. Analysis of sporulation by plasmid-cured strains.
Three plasmid-cured strains (SS292 2-2, 4-1 and 4-2), and one exconjugant still maintaining the plasmid as control (SS293), were grown on MYM agar for 4 days and analysed microscopically for sporulation by taking cover slip impressions. All exconjugants imaged displayed spores of equal size, indicating the absence of any sporulation defects. Scale bars 2 μm.

Assessment of plasmid-cured clones by whole-genome sequencing

Genomic DNA samples from two of the PCR-confirmed plasmid-cured candidates, denoted hereafter as SS-292-1 and SS-292-2, as well as the parental strain NRRL B-65442 and the more recently acquired type isolate ATCC 10712 as controls, were sequenced with Illumina (see methods above). Reads and contigs from each cured and control strain were mapped independently for each strain over the high-quality genome sequence for the parental strain NRRL B-65442 with BWA [24, 25] and the alignment processed with SAMtools [26] as previously described [40]; assembly files were visualised with BAMView [27] and their quality and coverage of replicons were assessed with Qualimap 2.2.1 [28, 29].

The following images (Fig. S5) show the coverage maps produced by Qualimap for each strain (from top to bottom, NRRL B-65442, ATCC 10712, SS-292-1, and SS-292-2). With NRRL B-65442 both reference replicons gave full coverage, with the plasmid having about the same coverage as the chromosome. For ATCC 10712 both reference replicons were also fully covered by the Illumina data, but with a much higher coverage for the plasmid. For both plasmid-cured strains, while the chromosome reference sequence was fully covered from end to end, the lack of a significant number of reads mapping to the plasmid reference sequence confirmed the loss of the complete plasmid. Table S5 summarises the relevant numbers represented by the coverage plots.

Figure S5. Qualimap analysis of Illumina data.

Coverage maps of Illumina reads from each strain over NRRL B-65442 as reference. From top, NRRL B-65442, ATCC 10712, SS-292-1, and SS-292-2

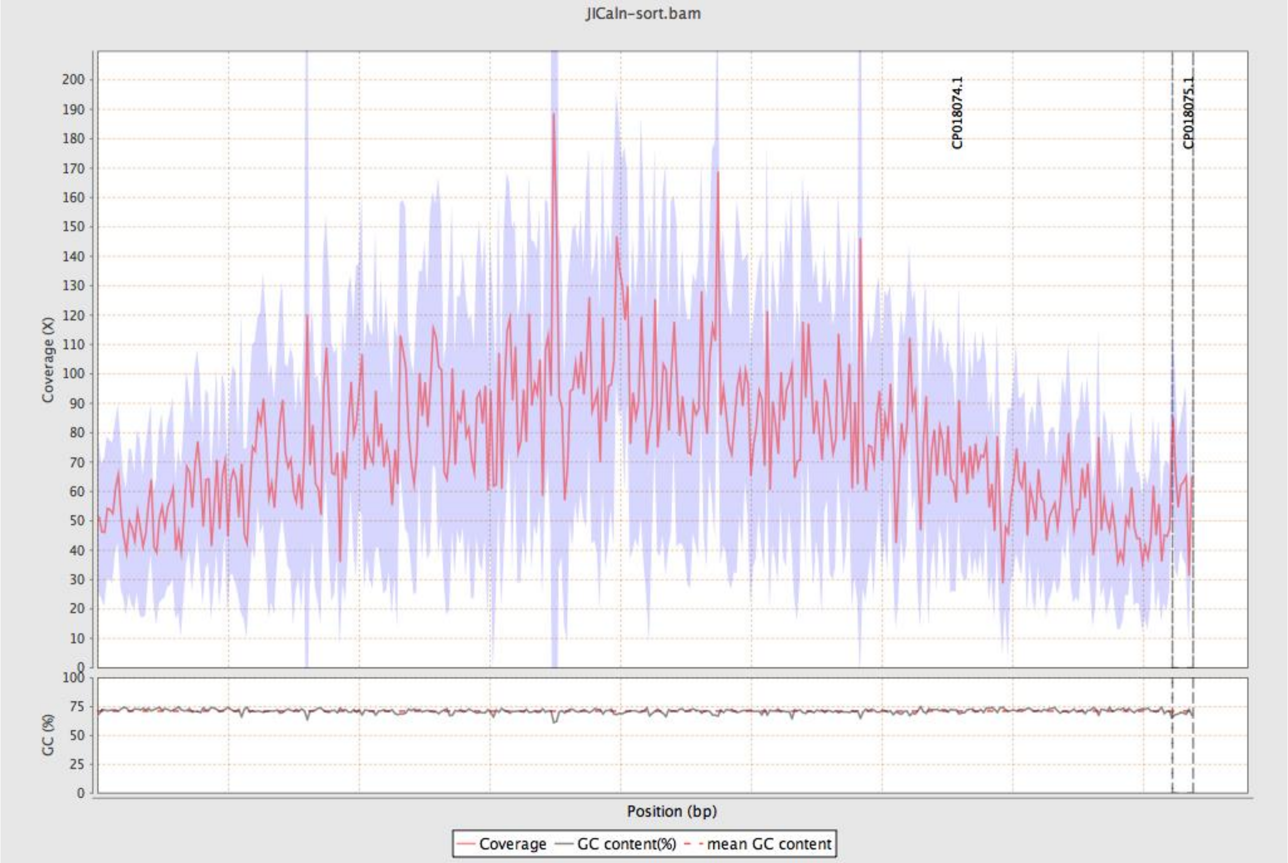

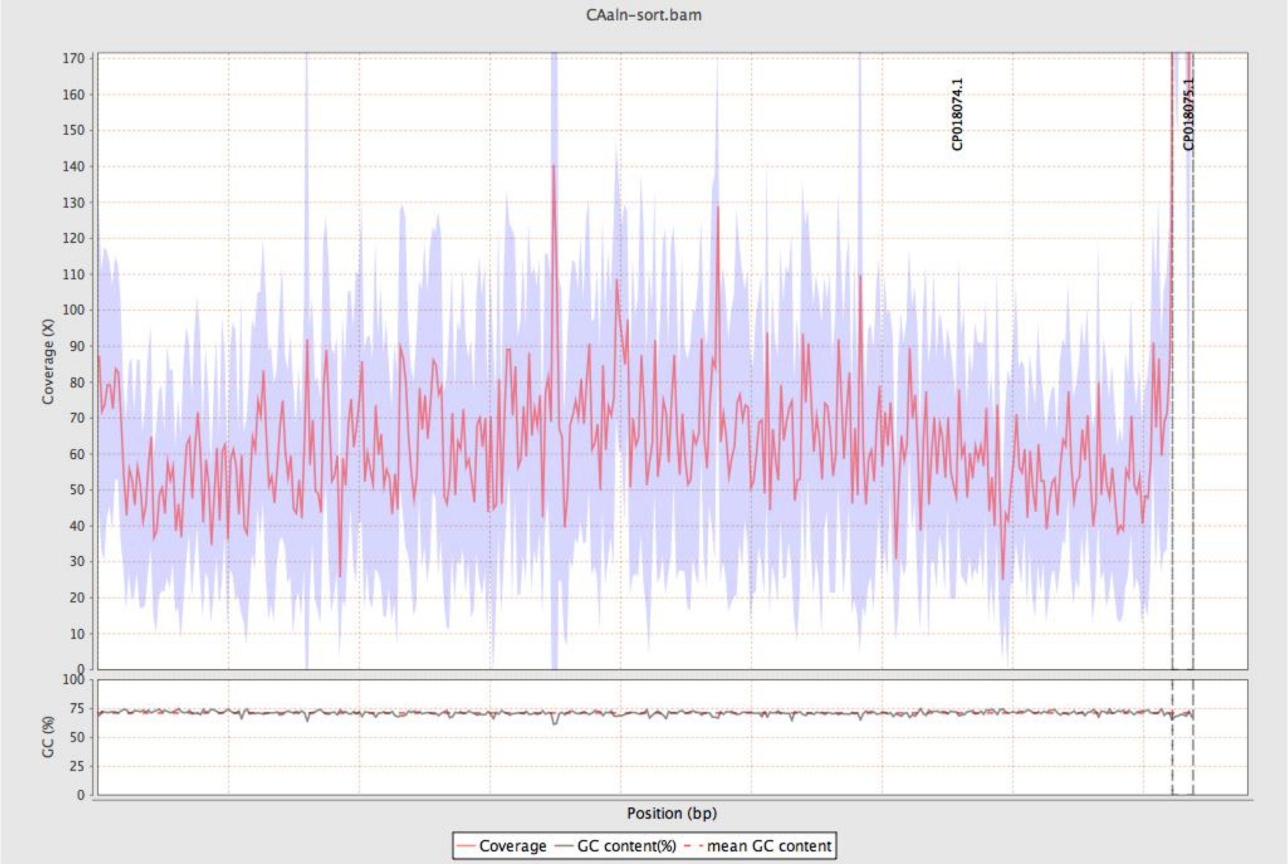

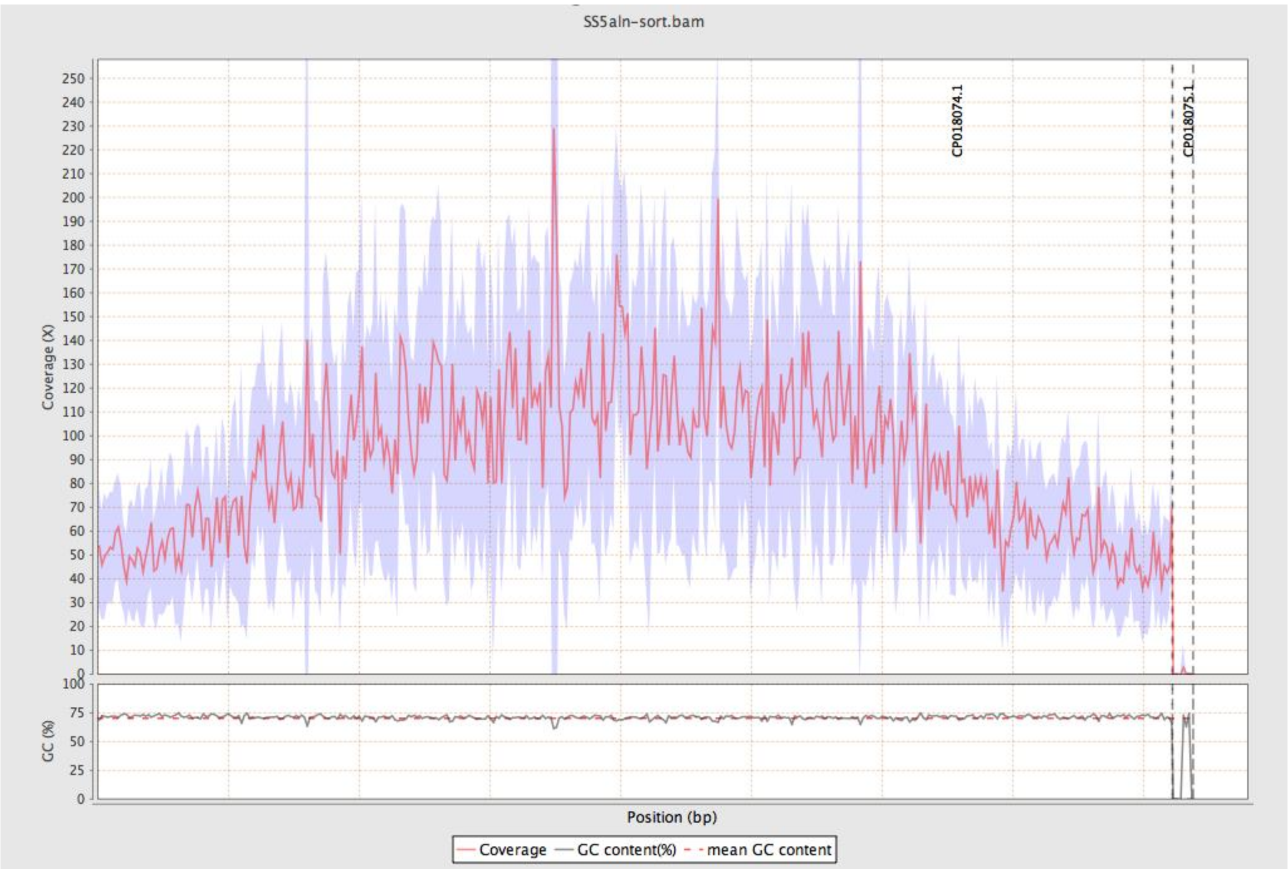
SS5aln-sort.bam
Coverage (X)
CP018074.1
CP018075.1
GC (%)
Position (bp)
Coverage
GC content(%)
mean GC content

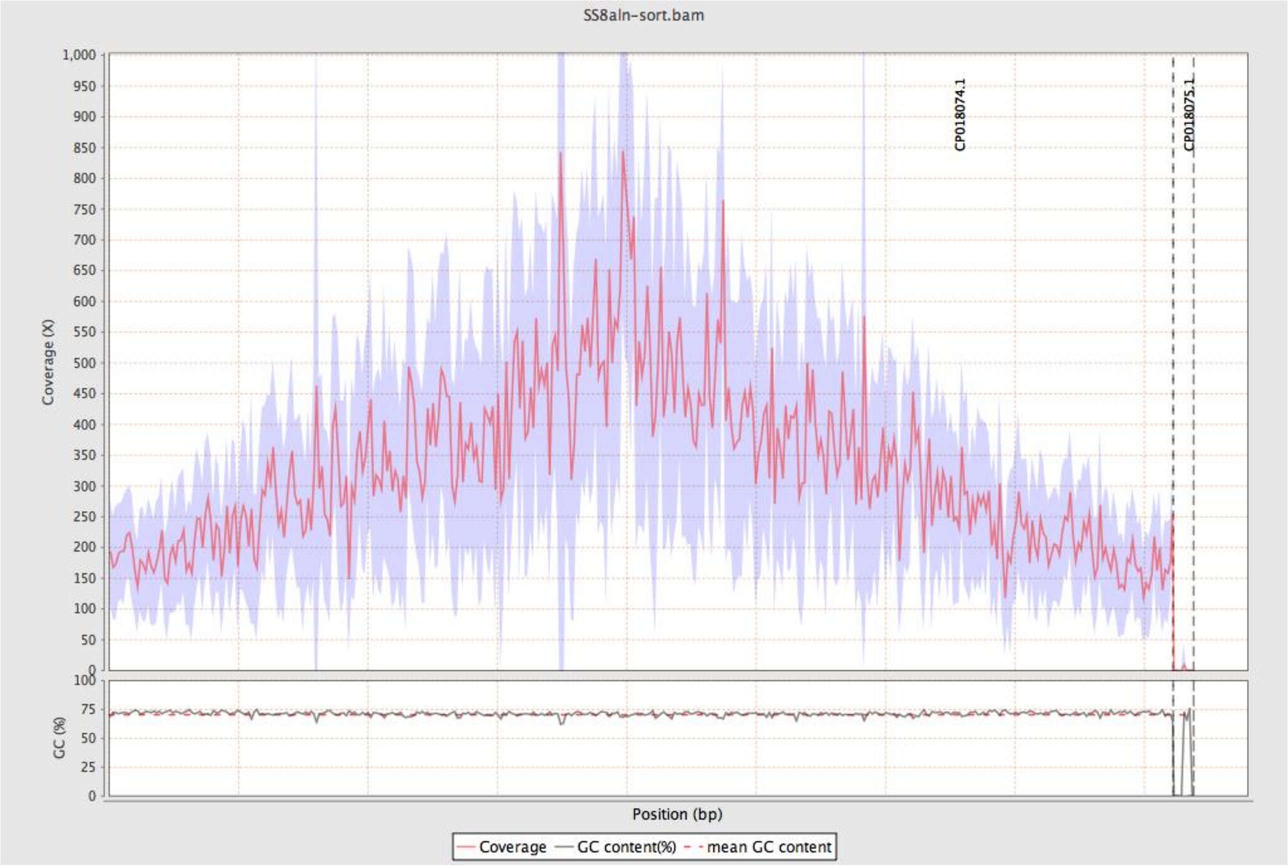
SS8aln-sort.bam
Coverage (X)
CP018074.1
CP018075.1
GC (%)
Position (bp)
Coverage
GC content(%)
mean GC content

Table S5. Coverage of each replicon by Illumina reads
(data from Qualimap report).

S. venezuelae strain	NRRL B-65442	ATCC 10712	SS-292-1	SS-292-2
Mean Coverage	75.635	67.0838	88.3011	325.3573
Standard Deviation Coverage	49.8807	54.797	59.8426	236.6972
Mean Mapping Quality	216.83	217.31	209.95	213.67
**Mean coverage per replicon**				
Chrm_CP018074.1	75.928	62.6389	89.9917	331.5904
plasmid_CP018075.1	60.3987	298.2131	0.3906	1.2424
**Coverage times chromosome**				
Chrm_CP018074.1	1	1	1	1
plasmid_CP018075.1	0.8	4.8	0.004	0.004
**Standard deviation coverage per replicon**				
Chrm_CP018074.1	50.1538	40.0462	59.1463	234.6089
plasmid_CP018075.1	28.8258	145.9792	3.6689	12.301

Extension of the ends of the replicons and identification of putative chromosomal terminal inverted repeats and a potential telomere structure

During the analysis of the plasmid-cured isolates (performed during 2019 and 2020) and using the parental strain as control, we obtained an Illumina assembly that, despite being very fragmented, provided additional sequence information at the ends of the chromosome and plasmid. Illumina contigs were selected by blastN searches with the ends of the current assembly; then all of the contigs were aligned to the current assembly to assess their reliability and overall alignment. The chromosome sequence was extended 601 bp at the left end and 752 bp at the right end (8223551 bp in total), while the plasmid sequence was extended 105 bp and 42 bp at the left and right ends, respectively (158269 bp in total); these additional sequences are provided below independently of the submitted assembly.

This extension, though short, allowed us to identify the putative start of the chromosomal terminal inverted repeats (TIR) typical of *Streptomyces* linear replicons; a blastN alignment performed at NCBI with the "752bp_Chromosome_extension_downstream_CP018074.1" sequence below as query and "CP018074.1" as subject revealed a 348 bp repeated and inverted sequence, from nucleotide 407 to 752 of the query sequence and nucleotide 927 to 581 of the subject sequence (i.e. the reverse-complement) with 93.7% identity; this indicates that the TIR runs for over 1.5 kb (927bp of the 2016 chromosome plus the additional 601 bp of new sequence).

This would be the first time that the TIRs of *S. venezuelae* had been identified. Furthermore, we identified putative palindromes (with https://www.bioinformatics.nl/cgi-bin/emboss/palindrome) with the potential to form potential secondary structures of the type previously described for *Streptomyces* telomers [41, 42, 43] (structure obtained with http://rna.tbi.univie.ac.at//cgi-bin/RNAWebSuite/RNAfold.cgi [44] for the first 200 nucleotides of "601bp_Chromosome_extension_upstream_CP018074.1") :

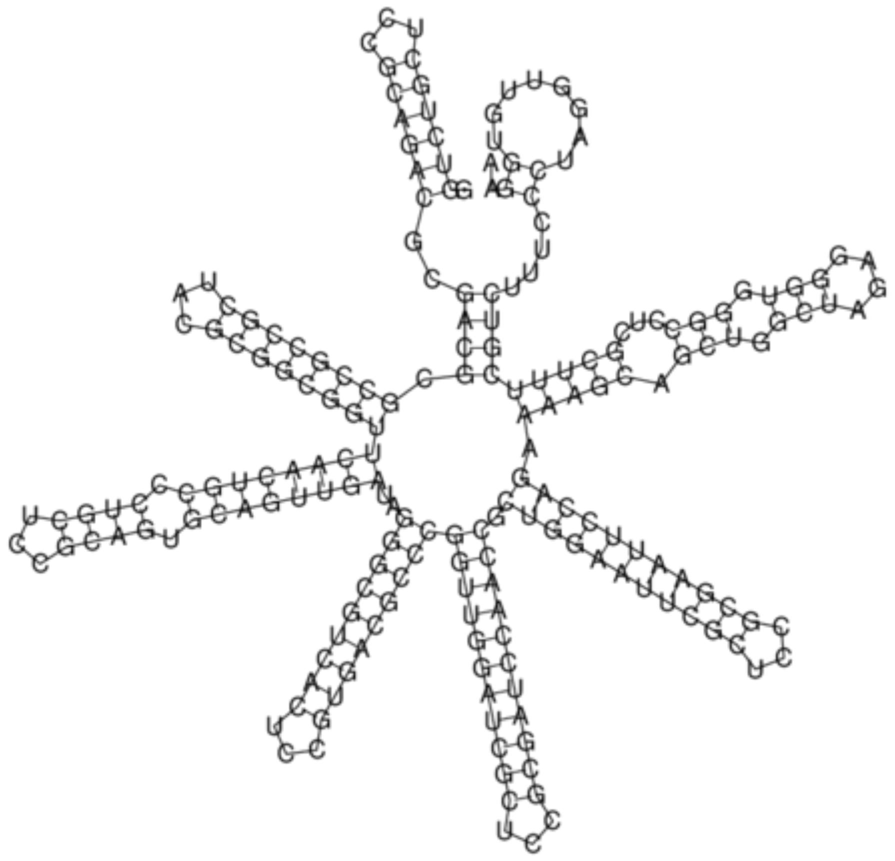

Sequences extending the ends of the 2016 chromosome and plasmid

```
>601bp_Chromosome_extension_upstream_CP018074.1
GGTCTGCTCCGCAGACGCGACGCGCCGCCGCTACGCGGCGGTTCAACTGC
CCTGCTCCGCAGTGCAGTTGATAGGGCGTCACTCCGTGACGCCCGGTTGG
ATCGCTCCGCGATCCAACCGCTGGAATTCGCTCCGCGAATTCCAGAAAAG
CAGCTGGCTAGAGGGTGGGCCTCGCTTTCGTCTTTCCCTAGGTTGTGGAA
TTCGCAATTAATTCTCATTGGTTGCGAATTACCAAAGAATGCGAAATATC
TATTTCCATGTTCTCGTGGAACTTTCAACAGCCCGTGCCGGTGCAAAAGG
TGAGTTTGTATCAGGGGTTCCGTGGCTGTCTCTGTGTGCCCGAGATCGCT
GAGGCCGGCGCCTCCCCCTCACGGCACGATGGCCTCCGAGCGCGGCGTGG
GGCCGACCTTGAGCATCGCCTCCACCCGGAGCATGGGCTGTCGTCTCTGC
TCACCTTGAGCGGATTGCCCCCAGAGCCTTCACGCCGTCAGGAACGGGAT
AGTGGCGGGGCGTTCGTGGTCCCTGGGGCCCGGTCGACGAGCGGGCTTGT
CCCCTGCCGGGCTGGTGCTTCTGGTGGTCGGGCCCGGCCGGGTGGTCAGG
C

>752bp_Chromosome_extension_downstream_CP018074.1
GCTTTTCGAGAAGGGCCGTTCCGTCTTTACGGAGCTCCATGCGGGTCCCC
TCGACGTTCTCCCAGCCCCCAGCGGCTTCGGCCGCCGTGGCGTCGTGGAT
CCCTGTTCCCTCGTAGTACTGGTAGGGGCCGGCGCACCCTGTCAGAGACG
CCGCACCCACTATCAAGACACCAAGGACCGCCCGCCACGCACACAGCCTG
CGTCCGCCTGCCACCAGGACCTCCCACCCTCAGCCCGTATCGGCCAACCG
ATAGGAGCAGCCGTGGAGAGATACCCTGCCAGACATGTTGAACGCGTTCA
ACTCTGCGGCTGAAGAGGTGGCGGTCACCCTCACAACTCGTGCGTACAGC
GCTGCCCGCAACCGCCGACAAGCACCATGAACAGCGTTCTCGATACAGGG
CCTAGACCGCGCTTCGGGCGTCGGAACGCTCCCGAGGTGCAGCCTTGCAT
```

```
CGCCGGCTGCACCCTTGGGTGGCCTGTGTCCCTTTCGGCGCTTCGAGGCC
TGGCTTGCTGTCGGGTGCGTTGTTGGCGGGTGGGTTGGCGGGTCCGTTCT
GGTTGTTTCGGTGTGGCTGGTTTCTGTCTCCGGGATTGTGTCCGTTCTCT
TGGTGTCAGGAGTTTTGGACGGGGGGAGTGAATTCTCTTTCTGTTCGGGT
GATTTGACGGGTTGTCTTTGATTTGCGTTGGGGGAGCTGGTATTTTGGGG
TGTAGGTATTTTCGAGTTGCTCTCGTTGTGGGGGCGGGTTAGTGGTGGAT
GG

>105bp_Plasmid_extension_upstream_CP018075.1
GGGTCCCATCGCGCTGCGCGCGATGCAAGCGAACCCCCACGCTGCGCGTG
GGGGTTGCGCTCCCGCTCCGCAGGAGCGCTAGCGGGGGGCTCCGCCTCCC
GCTCA

>42bp_Plasmid_extension_downstream_CP018075.1
GCGGGTGTTCGCTTGCACATCGCGCAGCGATGTGGTACCCGC
```

REFERENCES

1. **Stuttard C**. Temperate phages of *Streptomyces venezuelae*: Lysogeny and host specificity shown by phages SV1 and SV2. *Microbiology* 1982;128:115–121, doi:10.1099/00221287-128-1-115.

2. **Kieser T, Bibb MJ, Buttner MJ, Chater KF, Hopwood DA**. *Practical Streptomyces genetics*. Norwich: The John Innes Foundation; 2000 ISBN 978-0-7084-0623-6.

3. **Sambrook J, Fritsch EF, Maniatis T**. *Molecular cloning: a laboratory manual*. Cold Spring Harbor, N.Y.: Cold Spring Harbor Laboratory; 1989 ISBN 978-0-87969-309-1.

4. **Ehrlich J, Bartz QR, Smith RM, Joslyn DA, Burkholder PR**. Chloromycetin, a new antibiotic from a soil actinomycete. *Science* 1947;106:417–417, doi:10.1126/science.106.2757.417.

5. **Smadel JE**. Chloramphenicol (chloromycetin) in the treatment of infectious diseases. *The American Journal of Medicine* 1949;7:671–685, doi:10.1016/0002-9343(49)90389-7.

6. **Ehrlich J, Gottlieb D, Burkholder PR, Anderson LE, Pridham TG**. *Streptomyces venezuelae*, N. Sp., the source of chloromycetin. *J Bacteriol* 1948;56:467–477.

7. **Ehrlich J, Smith RM, Penner MA**. *Process for the manufacture of chloramphenicol*. US2483892A.

8. **Carter HE, Gottlieb D, Anderson HW**. Chloromycetin and streptothricin. *Science* 1948;107:113–113, doi:10.1126/science.107.2770.113-b.

9. **Stuttard C**. Transduction of auxotrophic markers in a chloramphenicol-producing strain of *Streptomyces*. *Microbiology* 1979;110:479–482, doi:10.1099/00221287-110-2-479.

10. **Doull J, Ahmed Z, Stuttard C, Vining LC**. Isolation and characterization of *Streptomyces venezuelae* mutants blocked in chloramphenicol biosynthesis. *Microbiology,* 1985;131:97–104, doi:10.1099/00221287-131-1-97.

11. **Ahmed ZU, Vining LC**. Evidence for a chromosomal location of the genes coding for chloramphenicol production in *Streptomyces venezuelae*. *J Bacteriol* 1983;154:239–244.

12. **Vining LC, Westlake DWS**. Biosynthesis of the phenylpropanoid moiety of chloramphenicol. *Can J Microbiol* 1964;10:705–716, doi:10.1139/m64-090.

13. **Malik VS, Vining LC**. Metabolism of chloramphenicol by the producing organism. *Can J Microbiol* 1970;16:173–179, doi:10.1139/m70-030.

14. **Malik VS, Vining LC**. Chloramphenicol resistance in a chloramphenicol-producing *Streptomyces*. *Can J Microbiol* 1972;18:583–590, doi:10.1139/m72-092.

15. **Malik VS, Vining LC**. Effect of chloramphenicol on its biosynthesis by *Streptomyces* species 3022a. *Can J Microbiol* 1972;18:137–143, doi:10.1139/m72-023.

16. **Stuttard C, Dwyer M**. A new temperate phage of *Streptomyces venezuelae*: morphology, DNA molecular weight, and host range of SV2. *Can J Microbiol* 1981;27:496–499, doi:10.1139/m81-073.

17. **Malpartida F, Hallam SE, Kieser HM, Motamedi H, Hutchinson CR, *et al.*** Homology between *Streptomyces* genes coding for synthesis of different polyketides used to clone antibiotic biosynthetic genes. *Nature* 1987;325:818–821, doi:10.1038/325818a0.

18. **Francis MM, Cella R, Vining LC**. Genetic recombination in a chloramphenicol–producing strain of *Streptomyces* species 3022a. *Can J Microbiol* 1975;21:1151–1159, doi:10.1139/m75-172.

19. **He J, Magarvey N, Piraee M, Vining LC**. The gene cluster for chloramphenicol biosynthesis in *Streptomyces venezuelae* ISP5230 includes novel shikimate pathway homologues and a monomodular non-ribosomal peptide synthetase gene. *Microbiology,* 2001;147:2817–2829, doi:10.1099/00221287-147-10-2817.

20. **Pullan ST, Chandra G, Bibb MJ, Merrick M**. Genome-wide analysis of the role of GlnR in *Streptomyces venezuelae* provides new insights into global nitrogen regulation in actinomycetes. *BMC Genomics* 2011;12:175, doi:10.1186/1471-2164-12-175.

21. **Gomez-Escribano Juan Pablo, Bibb Mervyn J.** Engineering *Streptomyces coelicolo*r for heterologous expression of secondary metabolite gene clusters. *Microbial Biotechnology* 2010;4:207–215, doi:10.1111/j.1751-7915.2010.00219.x.

22. **Rutherford K, Parkhill J, Crook J, Horsnell T, Rice P, *et al.*** Artemis: sequence visualization and annotation. *Bioinformatics* 2000;16:944–945, doi:10.1093/bioinformatics/16.10.944.

23. **Carver TJ, Rutherford KM, Berriman M, Rajandream M-A, Barrell BG, *et al.*** ACT: the Artemis comparison tool. *Bioinformatics* 2005;21:3422–3423, doi:10.1093/bioinformatics/bti553.

24. **Li H, Durbin R**. Fast and accurate short read alignment with Burrows-Wheeler transform. *Bioinformatics* 2009;25:1754–1760, doi:10.1093/bioinformatics/btp324.

25. **Li H, Durbin R**. Fast and accurate long-read alignment with Burrows–Wheeler transform. *Bioinformatics* 2010;26:589–595, doi:10.1093/bioinformatics/btp698.

26. **Li H, Handsaker B, Wysoker A, Fennell T, Ruan J, *et al.*** The Sequence Alignment/Map format and SAMtools. *Bioinformatics* 2009;25:2078–2079, doi:10.1093/bioinformatics/btp352.

27. **Carver T, Bohme U, Otto TD, Parkhill J, Berriman M**. BamView: viewing mapped read alignment data in the context of the reference sequence. *Bioinformatics* 2010;26:676–677, doi:10.1093/bioinformatics/btq010.

28. **García-Alcalde F, Okonechnikov K, Carbonell J, Cruz LM, Götz S, *et al.*** Qualimap: evaluating next-generation sequencing alignment data. *Bioinformatics* 2012;28:2678–2679, doi:10.1093/bioinformatics/bts503.

29. **Okonechnikov K, Conesa A, García-Alcalde F**. Qualimap 2: advanced multi-sample quality control for high-throughput sequencing data. *Bioinformatics* 2016;32:292–294, doi:10.1093/bioinformatics/btv566.

30. **Staden R, Beal KF, Bonfield JK**. The Staden Package, 1998. In: Misener S, Krawetz SA (editors). *Bioinformatics Methods and Protocols*. Totowa, NJ: Humana Press. pp. 115–130 ISBN 978-1-59259-192-3.

31. **Bonfield JK, Whitwham A**. Gap5—editing the billion fragment sequence assembly. *Bioinformatics* 2010;26:1699–1703, doi:10.1093/bioinformatics/btq268.

32. **Altschul SF, Madden TL, Schäffer AA, Zhang J, Zhang Z, *et al.*** Gapped BLAST and PSI-BLAST: a new generation of protein database search programs. *Nucleic Acids Res* 1997;25:3389–3402, doi:10.1093/nar/25.17.3389.

33. **Santiago-Sotelo P, Ramirez-Prado JH**. prfectBLAST: a platform-independent portable front end for the command terminal BLAST+ stand-alone suite. *BioTechniques* 2012;53:299–300, doi:10.2144/000113953.

34. **Aziz RK, Bartels D, Best AA, DeJongh M, Disz T, *et al.*** The RAST Server: Rapid Annotations using Subsystems Technology. *BMC Genomics* 2008;9:75, doi:10.1186/1471-2164-9-75.

35. **Overbeek R, Olson R, Pusch GD, Olsen GJ, Davis JJ, *et al.*** The SEED and the Rapid Annotation of microbial genomes using Subsystems Technology (RAST). *Nucl Acids Res* 2014;42:D206–D214, doi:10.1093/nar/gkt1226.

36. **Blin K, Wolf T, Chevrette MG, Lu X, Schwalen CJ, *et al.*** antiSMASH 4.0-improvements in chemistry prediction and gene cluster boundary identification. *Nucleic Acids Res* 2017;45:W36–W41, doi:10.1093/nar/gkx319.

37. **Cobb RE, Wang Y, Zhao H**. High-efficiency multiplex genome editing of *Streptomyces* species using an engineered CRISPR/Cas system. *ACS Synth Biol* 2015;4:723–728, doi:10.1021/sb500351f.

38. **Wang Y, Cobb RE, Zhao H**. Chapter Twelve - High-efficiency genome editing of *Streptomyces* species by an engineered CRISPR/Cas system. In: O'Connor SE (editor). *Methods in Enzymology*. Academic Press. pp. 271–284.

39. **Schlimpert S, Flärdh K, Buttner M**. Fluorescence time-lapse imaging of the complete *S. venezuelae* life cycle using a microfluidic device. *JoVE* 2016;53863, doi:10.3791/53863.

40. **Gomez-Escribano JP, Castro JF, Razmilic V, Chandra G, Andrews B, *et al.*** The *Streptomyces leeuwenhoekii* genome: *de novo* sequencing and assembly in single contigs of the chromosome, circular plasmid pSLE1 and linear plasmid pSLE2. *BMC Genomics* 2015;16:485, doi:10.1186/s12864-015-1652-8.

41. **Tidjani AR, Bontemps C, Leblond P.** Telomeric and sub-telomeric regions undergo rapid turnover within a *Streptomyces* population. Sci Rep. 2020;10, 7720. https://doi.org/10.1038/s41598-020-63912-w.

42. **Huang C-H, Lin Y-S, Yang Y-L, Huang S, Chen CW**. The telomeres of *Streptomyces* chromosomes contain conserved palindromic sequences with potential to form complex secondary structures. *Molecular Microbiology* 1998;28:905–916, doi:10.1046/j.1365-2958.1998.00856.x.

43. **Yang C-C, Tseng S-M, Pan H-Y, Huang C-H, Chen CW**. Telomere associated primase Tap repairs truncated telomeres of *Streptomyces*. *Nucleic Acids Res* 2017;45:5838–5849, doi:10.1093/nar/gkx189.

44. **Gruber AR, Lorenz R, Bernhart SH, Neuböck R, Hofacker IL**. The Vienna RNA Websuite. *Nucleic Acids Research* 2008;36:W70–W74, doi:10.1093/nar/gkn188.
